# ATR Kinase Is a Crucial Player Mediating the DNA Damage Response in *Trypanosoma brucei*

**DOI:** 10.3389/fcell.2020.602956

**Published:** 2020-12-22

**Authors:** Paula Andrea Marin, Ricardo Obonaga, Raphael Souza Pavani, Marcelo Santos da Silva, Christiane Bezerra de Araujo, André Arruda Lima, Carla Cristi Avila, Igor Cestari, Carlos Renato Machado, Maria Carolina Elias

**Affiliations:** ^1^Laboratory of Cell Cycle (LCC), Center of Toxins, Immune Response and Cell Signaling (CETICs), Butantan Institute, São Paulo, Brazil; ^2^Institute of Parasitology, McGill University, Montreal, QC, Canada; ^3^Division of Experimental Medicine, McGill University, Montreal, QC, Canada; ^4^Biochemical and Immunology Department, Institute of Biomedical Science, Federal University of Minas Gerais, Belo Horizonte, Brazil

**Keywords:** ATR, DNA damage response, checkpoint, γH2A, RAD51, *Trypanosoma brucei*, DNA damage response, DNA double-strand breaks

## Abstract

DNA double-strand breaks (DSBs) are among the most deleterious lesions that threaten genome integrity. To address DSBs, eukaryotic cells of model organisms have evolved a complex network of cellular pathways that are able to detect DNA damage, activate a checkpoint response to delay cell cycle progression, recruit the proper repair machinery, and resume the cell cycle once the DNA damage is repaired. Cell cycle checkpoints are primarily regulated by the apical kinases ATR and ATM, which are conserved throughout the eukaryotic kingdom. *Trypanosoma brucei* is a divergent pathogenic protozoan parasite that causes human African trypanosomiasis (HAT), a neglected disease that can be fatal when left untreated. The proper signaling and accuracy of DNA repair is fundamental to *T. brucei* not only to ensure parasite survival after genotoxic stress but also because DSBs are involved in the process of generating antigenic variations used by this parasite to evade the host immune system. DSBs trigger a strong DNA damage response and efficient repair process in *T. brucei*, but it is unclear how these processes are coordinated. Here, by knocking down ATR in *T. brucei* using two different approaches (conditional RNAi and an ATR inhibitor), we show that ATR is required to mediate intra-S and partial G1/S checkpoint responses. ATR is also involved in replication fork stalling, is critical for H2A histone phosphorylation in a small group of cells and is necessary for the recruitment and upregulation of the HR-mediated DNA repair protein RAD51 after ionizing radiation (IR) induces DSBs. In summary, this work shows that apical ATR kinase plays a central role in signal transduction and is critical for orchestrating the DNA damage response in *T. brucei*.

## Introduction

DNA double-strand breaks (DSBs) are among the most toxic forms of DNA damage that threaten genomic integrity. It can be induced via the effect of cellular metabolites or by DNA-damaging agents (e.g., ionizing radiation) (van Gent et al., 2001). When DSBs are not properly repaired, chromosomal rearrangements, deletions and even cell death can be the result (Khanna and Jackson, 2001; Jackson and Bartek, 2009). To maintain genomic integrity, the eukaryotic cells of model organisms have a complex evolutionarily conserved network of cellular pathways known as the DNA damage response (DDR) that orchestrates the detection and repair of a wide range of DNA damage (Zhou and Elledge, 2000; Harper and Elledge, 2007; Jackson and Bartek, 2009). DDR usually involves the specific recognition of DNA damage, followed by signal transduction and activation of effector molecules. Additionally, the DDR activates a checkpoint response that culminates in cell cycle arrest or a delay cell cycle progression, providing enough time for DNA repair before the cell enters the next cell cycle phase (Zhou and Elledge, 2000; Harper and Elledge, 2007). Once DNA damage is repaired, the cell cycle is resumed.

In model organisms, DDR is mainly controlled by ataxia telangiectasia mutated (ATM) and ataxia telangiectasia and Rad3-related (ATR), two members of the phosphoinositide 3-kinase (PI3K)-related kinase (PIKK) protein kinase family (Lempiainen and Halazonetis, 2009; Lovejoy and Cortez, 2009), which act together to orchestrate DNA repair and maintain genome integrity. In response to DNA damage, these kinases are recruited and rapidly activated by specific cofactors (Zhou and Elledge, 2000), phosphorylating multiple substrates (Matsuoka et al., 2007). ATM is primarily activated by DSBs and is considered a master regulator of cellular responses to DSBs (Shiloh and Ziv, 2013). Although ATR is frequently associated with the replication stress response (Cimprich and Cortez, 2008), this kinase is involved in a wide range of DNA lesions that expose tracks of single-stranded DNA (ssDNA), including DSBs (Adams et al., 2006; Cuadrado et al., 2006; Jazayeri et al., 2006). ATR is recruited to tracts of ssDNA coated with the ssDNA binding protein complex, replication protein A (RPA) through its partner ATR-interacting protein (ATRIP) (Zou and Elledge, 2013). For its optimal activation, ATR requires the presence of ssDNA–double-stranded DNA (dsDNA) junctions and activator proteins such as topoisomerase-binding protein-1 (TOPBP1). The checkpoint clamp complex RAD9–RAD1–HUS1 (9-1-1) recognize ssDNA–dsDNA junctions and facilitate the recruitment of TOPBP1 through interaction that involve its binding to the C-terminal of the RAD9 subunit (Delacroix et al., 2007). Once TOPBP1 binds to damage site, it activates ATR in an ATRIP-dependent manner (Mordes et al., 2008). On the other hand, recent studies using *Xenopus* egg extracts have demonstrated that single strand break (SSB) end resection mediated by apurinic/apyrimidinic (AP) endonucleases such as APE2, can trigger ATR pathway following oxidative stress (Willis et al., 2013). The APE2-mediated SSB end resection generates ssDNA that stimulate the recruitment of ATR, ATRIP, TopBP1 and 9-1-1 complex onto damage site and activate ATR (Lin et al., 2018).

In contrast to ATM, ATR is essential in unperturbed proliferating cells (Brown and Baltimore, 2000; de Klein et al., 2000) and, together with its major downstream effector checkpoint kinase 1 (CHK1), can prevent excessive origin firing during the S phase (Marheineke and Hyrien, 2004; Katsuno et al., 2009; Saldivar et al., 2017). Furthermore, under replication stress, ATR and CHK1 are involved in the global suppression of origin firing, stabilization, repair, and reinitiation of the replication fork (Saldivar et al., 2017). Both ATR and ATM are involved in the regulation of cell cycle checkpoints typically active in the G1/S, intra-S, and G2/M phases. However, the activation of the intra-S phase and G2/M checkpoints are primarily related to ATR function, whereas the induction of the G1 cell cycle checkpoint is generally a function of the ATM kinase (Abraham, 2001).

DSBs generated in the G1 phase are repaired by non-homologous end-joining (NHEJ), and DSBs generated in the S and G2 phases are mainly repaired by homologous recombination (HR)-mediated repair mechanisms (Shrivastav et al., 2008). HR-mediated repair is initially promoted by ATM through the regulation of DNA-end resection (You et al., 2009; Bolderson et al., 2010), a process that generates tracts of the ssDNA required for homology searching and strand invasion mediated by RAD51 (Kowalczykowski, 2015). In response to DSBs, ATM is recruited to chromatin and activated by MRE11-RAD50-NBS1/XRS2 (MRN/X is MRN in humans and MRX in yeast), a complex that acts as a sensor of DSBs and is also critical for DNA-end resection initiation in conjunction with CtIP (Paull and Lee, 2005). Once recruited to the break site and activated, ATM phosphorylates S139 in the C-terminus of the histone variant H2AX (Rogakou et al., 1998) (referred to as γH2AX), forming the basis of a chromatin-based signaling cascade (Scully and Xie, 2013), which allows the recruitment of several DDR components (Celeste et al., 2002). In addition to H2AX, ATM also phosphorylates other substrates and stimulates DNA-end resection and HR (You et al., 2009; Bolderson et al., 2010). However, despite its role in promoting HR, ATM is not essential for HR-mediated repair, and this mechanism can occur in the absence of ATM (Rass et al., 2013).

In contrast to ATM, ATR seems to control the later steps of HR, and its inhibition or loss impairs the ability of cells to utilize HR (Kim et al., 2018). In this context, ATR can be activated by ssDNA intermediates formed by DBS processing, and while DNA end resection induces its activation, this same process also diminishes the capacity of dsDNA to activate ATM, switching from an ATM-activating mode to an ATR-activating mode during HR-mediated repair (Cuadrado et al., 2006; Shiotani and Zou, 2009). Additionally, ATR-CHK1 signaling enhances the capacity of cells to use HR-mediated repair by ensuring the proper level of expression of key factors in the HR machinery (Kim et al., 2018). ATR can also promote the recruitment of key HR factors required for strand invasion, such as PALB2 and BRCA2 (Buisson et al., 2017), and the stabilization of BRCA1 at DNA lesions via its interaction with TOPBP1, promoting DNA resection (Liu et al., 2017). All these findings indicate that ATR plays key roles in the regulation of HR-mediated repair.

The DSB response pathways are well characterized in model eukaryotes, while the understanding and characterization of these mechanisms in trypanosomatids are still in progress. *Trypanosoma brucei* is a eukaryotic protozoan parasite that causes human African trypanosomiasis (HAT), also known as sleeping sickness, which is fatal without therapy (Aksoy et al., 2017). In recent years, our knowledge of how *T. brucei* addresses DSBs has improved due to a better understanding of the antigenic variation induced by variant surface glycoprotein (VSG) switching, an efficient mechanism stimulated by DSBs that allows this parasite to evade the host immune system (Horn, 2014; da Silva M.S. et al., 2018). In this parasite, DSBs trigger a DNA damage response (Glover et al., 2008; Glover and Horn, 2009; Marin et al., 2018), which is repaired mainly by HR and microhomology-mediated end-joining (MMEJ) (Glover et al., 2008). NHEJ-mediated repair appears to be absent or has mechanistically diverged (Burton et al., 2007). Additionally, many of the main eukaryotic proteins involved in HR, such as H2A (Glover and Horn, 2012), MRE11 (Tan et al., 2002), RPA (Pavani et al., 2016; Marin et al., 2018; Glover et al., 2019), RAD51 (McCulloch and Barry, 1999), and BRCA2 (Hartley and McCulloch, 2008), have been identified in trypanosomatids, showing DDR responses similar to those of other eukaryotes.

Although many studies have explored DNA breaks in a VSG-switching context, little is known about the role of *T. brucei* ATR kinase in DSBs in general. Preliminary studies based on inducible RNAi knockdown of a *T. brucei* ATR kinase homolog showed that ATR loss leads to the impaired proliferation of the bloodstream form (BSF), cell cycle alteration and sensitization to genotoxic agents (Parsons et al., 2005; Jones et al., 2014; Black et al., 2020). Additionally, it has also been demonstrated that ATR can modulate antigenic variations through DNA damage signaling (Black et al., 2020). However, many proteins involved in ATR activation or downstream targets remain to be identified (Genois et al., 2014); for example ATRIP, an important ATR cofactor, Chk1 and Cdc25 family phosphatases involved in activation of ATR-mediated DNA damage checkpoint (Mailand et al., 2000; Sorensen et al., 2003). On the other hand, some homologs have been identified but have not been validated thus far, such as TopBP1, an important activator of ATR (Genois et al., 2014).

Here, using a tetracycline-controlled inducible RNAi expression system for ATR silencing and the ATR inhibitor VE-821 for knocking down ATR activity, we investigated the role of *T. brucei* ATR kinase in the DNA damage response to ionizing radiation-induced DSBs. Our findings indicate that ATR exhibits essential functions in controlling several processes within the DNA damage response in procyclic cells. *T. brucei* ATR is necessary for the proper progression through the cell cycle under unperturbed cell conditions, is required for mediating intra-S checkpoint activation and seems to contribute partially to G1/S checkpoint activation in response to IR-induced DSBs. We also found that ATR is involved in replication fork stalling in response to damage caused by IR. ATR also contributes to H2A histone phosphorylation (γH2A) and shows crucial functions in the recruitment and upregulation of RAD51 recombinase after IR irradiation. In summary, *T. brucei* ATR acts as an apical kinase critical for signal transduction and coordinates the DNA damage response to IR-induced DSBs.

## Results

### IdU and CldU Dual-Labeling Pulses Facilitate the Monitoring of Cell Progression Through the S Phase

**FIGURE 1:**
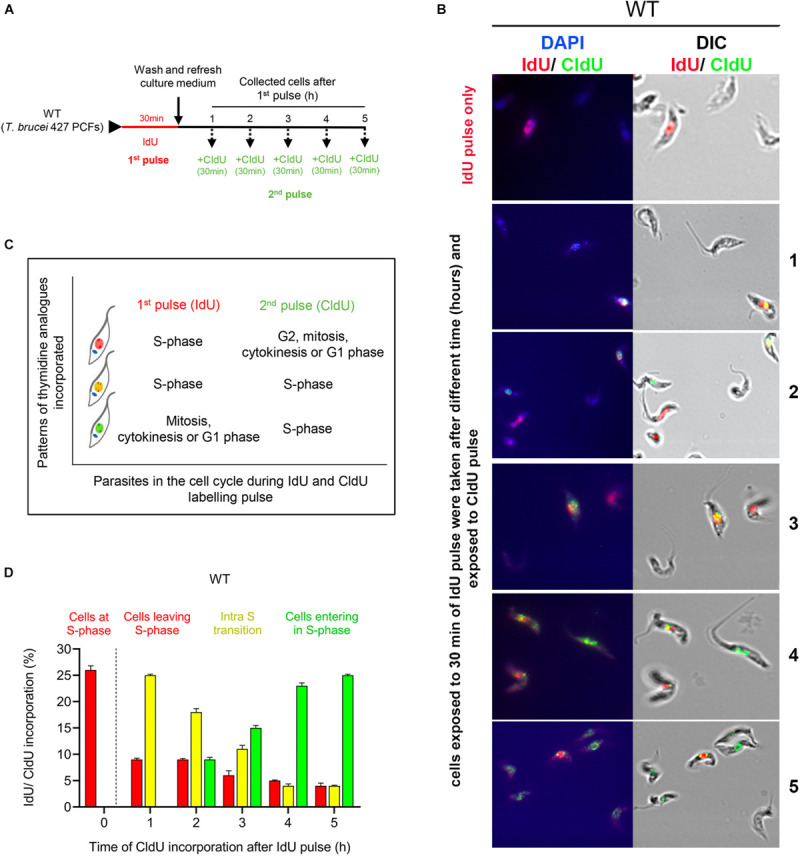
Detection of cell progression through the S phase using codetection of the incorporation of two thymidine analogs. **(A)** Scheme shows the experimental strategy for the detection of *T. brucei* PCFs that progress through the S phase by codetecting thymidine analog incorporation. Parasites were pulsed with IdU (red) for 30 min, washed and collected each hour for 5 h. Thirty minutes before each timepoint, the cells were pulse-labeled with CldU for 30 min. **(B)** Codetection of thymidine analogs in the WT cells. The incorporated analogs were immunodetected with specific antibodies and appeared red (IdU), green (CldU) or yellow (merged IdU and CldU). **(C)** Box relates the patterns of thymidine analog incorporation detected with cell progression through the S phase. **(D)** Graph shows the percentage of parasites with incorporated IdU, CldU or both at different time intervals. The data represent the average of three independent experiments, each consisting of *n* = 300, and the error bars represent the standard deviations.

To monitor cell progression through the S phase, we used an IdU and CldU dual-labeling strategy previously described and used in human cell lines (Seiler et al., 2007). Here, the thymidine analogs IdU and CIdU were sequentially incorporated into DNA in asynchronous cells according to the protocol shown in Figure 1A. Briefly, cells in the exponential growth stage were pulse-labeled with 5-Iodo-2′-deoxyuridine (ldU) for 30 min (1st pulse), washed, released in fresh medium and collected hourly for 5 h. Thirty minutes before each timepoint measurement (except point 0), the cells were pulse-labeled with 5-chloro-2′-deoxyuridine (CldU) for an additional 30 min (2nd pulse), fixed and examined by fluorescence microscopy using specific antibodies: anti-IdU (red) and anti-CldU (green) (Figure 1B and Supplementary Figures S1A,B). The cell collection time, hourly for 5 h, was established to analyze the activation and deactivation of the checkpoints during this period based on the time that *T. brucei* PCFs need to repair the DNA damage generated by a specific dose (50 Gy) of IR irradiation (Marin et al., 2018), a challenge condition that was included in subsequent experiments. Using this strategy, the first IdU pulse labeling allowed us to selectively identify cells in S phase at the beginning of the assay (red cells) (Figure 1B). The second CldU pulse labeling was used for three purposes: (i) to identify cells that were replicating in the first pulse and were still replicating (yellow cells, merged red and green fluorescence, classified as intra-S cells), (ii) to identify cells that were not replicating during the first pulse but entered the S phase at the established timepoints during the second pulse (labeled in green), and (iii) to identify cells that were in the S phase during the first pulse but exited the S phase during the second pulse (red). Figure 1C summarizes the patterns of thymidine analog incorporation.

**FIGURE 2:**
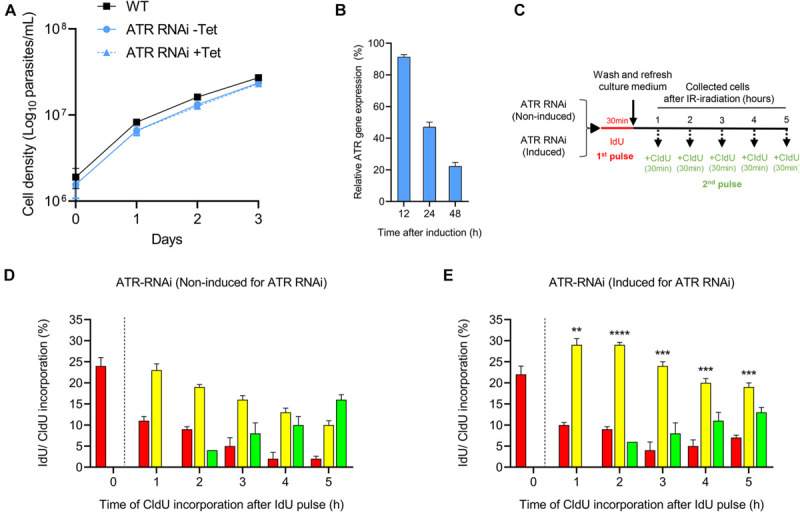
Role of ATR kinase in unperturbed cell progression through the S phase. **(A)** Curve plots show the viability of *T. brucei* PCFs before and after RNAi induction for ATR silencing with tetracycline (Tet). The data represent the averages of three independent experiments, and error bars the standard deviations. **(B)** RT-PCR analysis for the relative quantification of ATR transcripts after gene silencing by RNAi induction with tetracycline. **(C)** Scheme shows the experimental strategy used for the detection of parasites that progress through the S phase by detecting the thymidine analogs incorporated in non-induced and induced cells for 48 h for ATR silencing. **(D,E)** Bar plots representing the percentage of cells in each group that progressed through the S phase in the non-induced or induced population for 48 h for ATR silencing. Bar plot graphs show the average of three independent experiments, each consisting of *n* = 300. The means of the percentage of intra-S cells in the induced and non-induced populations after ATR silencing were compared, and significant differences were determined by *t-*test. Significance values are shown as ****P ≤ 0.0001, ***P ≤ 0.001, **P ≤ 0.01.

Incorporation analysis of thymidine analogs in the procyclic form (PCF) of wild-type (WT) *T. brucei* showed that after the first pulse, ∼26% of the cells had incorporated IdU as expected (Figure 1D, 0 h; red bar). One hour after the IdU pulse, ∼25% of the cells were synthesizing DNA, as demonstrated by the CldU incorporation in the cells that had previously incorporated the first analog (Figure 1D, 1 h; yellow bar). This percentage of intra-S phase cells gradually decreased over time from ∼18 to 11% from 2 to 3 h. From 4 and 5 h after IdU pulse, we continued detecting a small percentage of IdU-positive cells (<5%) incorporating CldU (Figure 1D, 4 and 5 h; yellow bars). The lower detection of IdU-positive cells at 3–5 h post-irradiation could be because part of the cells (those that incorporated less IdU because they were at the end of S phase) have undergone cell division (da Silva et al., 2017) and for this reason these could escape detection. Additionally, we also detected ∼10% of new cells entering the S phase starting 2 h after the first pulse (Figure 1D, 2 h; green bar), which gradually increased to ∼25% at 5 h (Figure 1D). Thus, we concluded that a dual-pulse labeling strategy with sequentially pulsed thymidine analogs, first with IdU and then with CldU at different time intervals, is a viable approach that can be used to monitor cell progression through the S phase.

### ATR Is Necessary for Proper Progression Through the S Phase

To examine the role of ATR in the control of cell progression through the S phase under normal culture conditions, we used *T. brucei* in PCF with a tetracycline-controlled inducible RNAi expression system for ATR silencing. First, we compared the cell proliferation rate of this cell line before and after 48 h of RNAi induction with the cell proliferation of the WT strain. We found that, even without RNAi induction, the population engineered for ATR silencing (the ATR RNAi population) showed a cell density that was slightly reduced compared with that of the WT strain (Figure 2A). On the other hand, RNAi induction did not lead to detectable changes in cell density compared to that shown by non-induced cells. However, reduced mRNA levels for ATR were detected 24 h after RNAi induction (Figure 2B). Next, we investigated whether ATR loss leads to perturbation of cell progression through the S phase. For this experiment, we pulsed the ATR RNAi population with IdU and CldU dual-labeling as described in Figure 2C. In the non-induced ATR RNAi population, the kinetics of the thymidine analog incorporation were similar to those of the WT population (Figures 1D, 2D), with cells actively replicating 1 h after the first pulse and the replication rate decreasing over time with new cells entering the S phase 2 h postexposure to IdU. No more than 10% of the cells were out of the S phase, a percentage that decreased over time (Figure 2D). After RNAi induction for ATR silencing, the kinetics of thymidine analog incorporation were comparable to those of the non-induced cells; however, the percentage of the cells progressing through the S phase (intra-S cells) was significantly higher compared to the percentage of the uninduced ATR RNAi population at all the times evaluated (1 h, 23.0% ± 1.5 vs. 29.0% ± 1.5, *P* ≤ 0.01; 2 h, 19 ± 0.6 vs. 29.0 ± 0.6, *P* ≤ 0.0001; 3 h, 16.0 ± 1.0 vs. 24.0 ± 1.0, *P* ≤ 0.001; 4 h, 13.0 ± 1.0 vs. 20.0 ± 1.0, *P* ≤ 0.001; and 5 h, 10.0 ± 1.0 vs. 19.0 ± 1.0, *P* ≤ 0.001) (Figures 2D,E). Thus, these results suggest that ATR is active and necessary for proper progression of unperturbed cells through the S phase.

### ATR Contributes to the Maintenance of Intra-S Checkpoint Activation in Response to IR-Induced DSBs

**FIGURE 3:**
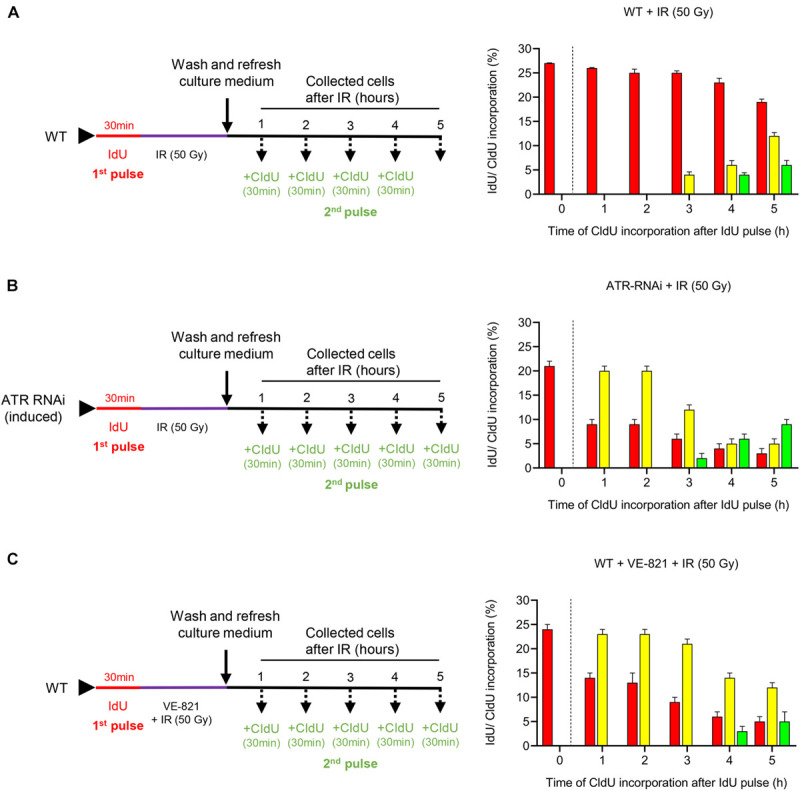
Functions of ATR in cell progression through the S phase. **(A–C)** Experimental strategy used (left) and the quantification (right) of the parasites that progress through the S phase in the WT, ATR-silenced and ATR-inhibited populations after IR irradiation. Bar plots show the percentage of parasites in each group (cells leaving, entering or in the intra-S phase) and kinetics of thymidine analog incorporation over time for each population after IR irradiation. **(A)** WT population was exposed to IdU for 30 min and then irradiated with 50 Gy of IR. Then, the cells were collected at the indicated times and pulsed with CIdU for 30 min before each measurement time. **(B)** Cells engineered to silence ATR (ATR RNAi) were induced 48 h before being exposed to IdU for 30 min. Then, the cells were pulsed with CIdU as in **(A)**. **(C)** WT population was pulsed with IdU as in **(A)**. Then, the cells were irradiated and cultured in the presence of the ATR inhibitor VE-821 and collected at predetermined times after CIdU pulse as in **(A)**. The data represent the average of three independent experiments, each consisting of *n* = 300, and the error bars represent the standard deviations.

Previously, we demonstrated that the treatment of the PCF of *T. brucei* with IR generates both DNA DSBs and a strong response to DNA damage (Marin et al., 2018). To analyze the checkpoint activation through the S phase in response to IR-induced DSBs and the effect on cell progression through this phase, we irradiated *T. brucei* in PCF with 50 Gy of IR and analyzed thymidine analog incorporation. For this experiment, WT cells were initially pulsed with IdU followed by irradiation with 50 Gy of IR. Then, the cells were collected at different time intervals without receiving a CldU pulse for 30 min before each measurement time, as described in Figure 3A (left) and then, anti-IdU and anti-CldU antibodies were used for immunodetection (Supplementary Figure S2A). The analysis of thymidine analog incorporation revealed that ∼27% of the cells were in S phase before irradiation, as indicated by the detection of incorporated IdU (Figure 3A, right). For the first 2 h after IR treatment, we detected only IdU-positive cells, and their percentage was similar to that of the non-irradiated population pulsed only with IdU, indicating that the cells that were in S phase at the time of the first IdU pulse had stopped replicating, probably due to the activation of an intra-S checkpoint in response to the damage caused by IR (Figure 3A, 1–2 h, right). From 3 to 5 h after irradiation, the percentage of IdU-labeled cells decreased slowly, while the cells that incorporated the two analogs were initially detected, demonstrating that the cells retained in S phase after irradiation had restored DNA synthesis and were transitioning through the S phase (Figure 3A, right). Additionally, new cells entering the S phase were detected only 4 h after irradiation, as determined by CldU incorporation, suggesting that in addition to intra-S checkpoint activation, another checkpoint between the G1/S transition was activated in response to IR damage (Figure 3A, right). Thus, these findings demonstrate that IR-induced DSBs stimulate strong intra-S and G1/S transition checkpoint activation in *T. brucei* cells.

Considering that ATR is involved in intra-S checkpoint control in model eukaryotes (Cimprich and Cortez, 2008; Saldivar et al., 2017), we wondered whether the ATR kinase of *T. brucei* would also have a conserved role in checkpoint activation control through the S phase in response to IR-induced DSBs. To assess this possibility, we induced ATR silencing in engineered *T. brucei* in the PCF that carried the tetracycline-controlled inducible RNAi expression system and subjected these cells to dual labeling with thymidine analogs by irradiating the cells after the first IdU pulse, as shown in Figure 3B (left), and then used immunodetection for the analysis (Supplementary Figure S2B). Before irradiation, the percentage of cells in this population in the S phase was similar to that detected in the non-irradiated WT population after subjection to only the IdU pulse (Figure 3B, right 0 h and Figure 1D, 0 h). However, in contrast to the irradiated WT population, in which the cells detected in S phase had stopped replicating within the first 2 h after IR and resumed replication only after 3 h (Figure 3A, right 1–5 h), the cells of the ATR RNAi population in S phase continued to replicate DNA actively during the first 2 h after irradiation, as demonstrated by the percentage of dual-labeled cells, which decreased after 3 h, showing S phase progression kinetics similar to those of the non-irradiated dual-pulsed WT cells (Figure 3B, right and Figure 1D). Additionally, this result was accompanied by a percentage of cells not in S phase that was similar to that of the non-irradiated WT cells at the times evaluated (Figure 3B, right and Figure 1D), indicating that ATR was necessary for the activation of the intra-S checkpoint. On the other hand, new cells entering the S phase were detected 1 h earlier than in the irradiated WT population (the values in Figure 3B, right vs. the values in Figure 3A, right, 3 h vs. 4 h), indicating that ATR can partially contribute to checkpoint activation during the G1/S transition.

On the other hand, taking advantage of the availability of the ATR inhibitor VE-821, which has previously been shown to inhibit the activity of this kinase selectively in trypanosomatids (da Silva R.B. et al., 2018), we treated WT cells with this inhibitor to compare the progression profile through the S phase with that found for the ATR-silenced population after irradiation. Before analyzing cell progression through the S phase, we subjected the WT parasites to different concentrations of VE-821 (1–50 μM) to identify the most suitable sublethal concentration that can inhibit cell growth over time without leading cells to death. The concentration that best matched our requirements was 5 μM (Supplementary Figure S3). For a cell progression analysis through the S phase, the parasites were exposed to the first pulse with IdU for 30 min. Then, the parasites were cultured in the presence of VE-821 and irradiated with 50 Gy of IR. Next, the cells were pulsed with CldU and collected at the times established, summarized in the protocol of presented in Figure 3C (left). Finally, the cells were visualized by immunofluorescence microscopy (Supplementary Figure S2C). The analysis of thymidine analogs incorporation indicated that the ATR-inhibited cell population showed kinetic cell progression through the S phase, similar to that of the ATR-silenced cells, in response to IR-induced DSBs (Figure 3C, right and Figure 3B, right). Additionally, we wondered whether ATM kinase of *T. brucei* would have any role on the DSB-induced intra-S and G1/S checkpoint response given its critical role as a master regulator of the cellular response to DSBs in higher eukaryotes (Shiloh and Ziv, 2013). For this purpose, we inhibited ATM kinase with the specific inhibitor KU55933. First, we evaluated the T. brucei cell growth in the presence of different concentrations of KU55933 (1–50 μM) and identify 20 μM of KU55933 as the most suitable sublethal concentration of ATM inhibitor (Supplementary Figure S4A). Then, the parasites were IdU pulsed for 30 min, washed, and cultured in the presence of the ATM inhibitor at the time of IR with 50 Gy. After that, the parasites were CIdU pulsed for 30 min before each measurement time and collected at the indicated times as summarized in Supplementary Figure S4B. Finally, the thymidine analogs incorporation was visualized by immunofluorescence microscopy (Supplementary Figure S4C). As observed in ATR-inhibited cell population, the cells treated with ATM inhibitor followed by IR did not arrest cell progression and, on the contrary, continued actively replicating through S phase (Supplementary Figure S4D). This finding suggests that, in addition to ATR, ATM also has a critical role in the activation of intra-S checkpoint followed by IR-induced damage as expected. Moreover, different to what was observed in WT and ATR-inhibited cell population, we detected an earlier entry of cells into S phase after IR (1 h vs. 3 or 4 h respectively), indicating that ATM could have a more critical function in G1/S checkpoint control compared to ATR.

### Silencing ATR or Loss of Its Activity Impairs Cell Cycle Progression

**FIGURE 4:**
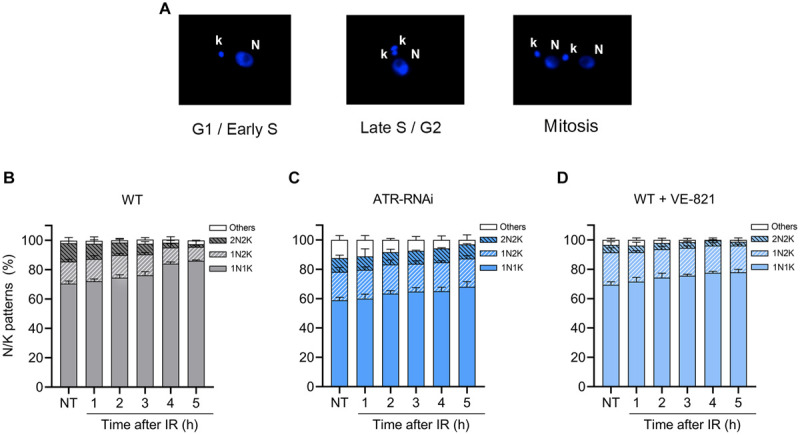
ATR loss or inhibition impairs cell cycle progression. **(A)** Representative nucleus (N) and kinetoplast (K) patterns after DAPI staining. **(B–D)** Measurement of N/K patterns in DAPI-stained parasites in WT, ATR-silenced and ATR-inhibited populations, respectively, before and after IR irradiation. The data represent the average of three independent experiments, each consisting of cells (*n* = 300). Error bars represent the standard deviations. Significant differences were determined by one-way ANOVA and Dunnett’s test for multiple comparisons (see text).

To investigate whether the loss of ATR can influence cell cycle progression under unperturbed conditions and after IR irradiation, we measured N/K patterns. This approach enables the determination of the percentage of cells in the G1/early-S phase, late-S/G2 phase, mitosis and atypical cell forms, as previously reported (Marin et al., 2018) and summarized in Figure 4A. The analysis of N/K patterns showed that in the non-irradiated WT population, ∼70% of the cells were in G1/early S, ∼15% of the cells were in late S/G2, ∼12% of the cells were undergoing mitosis, and < 2% of the cells were in other or atypical forms (Figure 4B, NT). Then, we analyzed these same N/K patterns in the ATR-silenced population and found that the percentage of cells in the G1/early-S phase was significantly lower (Figure 4B; WT, NT: 70.5% ± 1.80 vs. Figure 4C; ATR RNAi NT: 58.9% ± 2.0, *P* ≤ 0.001), while the percentage of atypical forms increased significantly compared to the WT population (Figure 4B; WT, NT: 1.8% ± 2.2 vs. Figure 4C; ATR RNAi, NT, 12.4% ± 3.0, *P* ≤ 0.01). On the other hand, the percentage of cells in mitosis was not significantly different with respect to the percentage of the cells in the WT population. Additionally, we also analyzed these cell cycle patterns in the population treated with VE-821; in this population, we also observed a decrease in the percentage of cells in the G1/early-S phase compared to the WT population, but this difference was not significant. In contrast to the ATR-silenced population, an increase in the percentage of cells in the late-S/G2 phase was found (Figure 4B; WT, 15.0% ± 1.7 vs. Figure 4D; ATRi, NT, 22.1% ± 3.0) and a significant decrease in the percentage of cells in mitosis compared to the WT population (Figure 4B; WT, 12.0% ± 1.6 vs. Figure 4D; ATRi, 5.1% ± 2.2). Together, these results suggest that ATR is necessary for proper progression through the cell cycle under normal culture conditions.

After the parasites were irradiated, we observed an accumulation of cells in G1/early S and a decrease of cells in mitosis in the WT population, reaching percentages of 86% and < 2%, respectively, at 5 h (Figure 4B), suggesting the activation of the G1/S transition checkpoint after irradiation, as previously observed and shown in Figure 3B. In the irradiated population with ATR silencing, we also observed a leaky accumulation of cells in G1/early-S phases during the 5 h postirradiation (Figure 4C). However, this was difference was statistically low compared to the percentage of cells of the WT population in G1/early S at all points evaluated (Figures 4B,C). Additionally, the percentage of cells in mitosis did not decrease as in the case of the WT population and was statistically higher 4 and 5 h after irradiation (4 h, WT: 9.5% ± 0.46 vs. ATR RNAi: 3.3% ± 1.5; 5 h, WT: 9.6% ± 0.5 vs. ATR RNAi: 1.8% ± 2.2, *P* ≤ 0.001) (Figures 4B,C). In cells treated with ATR inhibitor, these alterations in the cell cycle after irradiation were less noticeable (Figure 4D). However, as with ATR silenced cells, the accumulation in G1/early-S cells 4 and 5 h after irradiation was statistically low compared to that of the WT cells (4 h; WT: 84.01% ± 1.20 vs. ATR RNAi: 77.60% ± 1.00, 5 h; WT: 86% ± 0.57 vs. ATR RNAi: 78.00% ± 2.00, *P* ≤ 0.01) (Figures 4B,D). These results support the hypothesis that ATR may have a function in G1/S checkpoint control, as previously suggested.

### ATR Kinase Is Necessary for Stalling and Stabilizing the Replication Fork After DNA Damage Caused by IR

**FIGURE 5:**
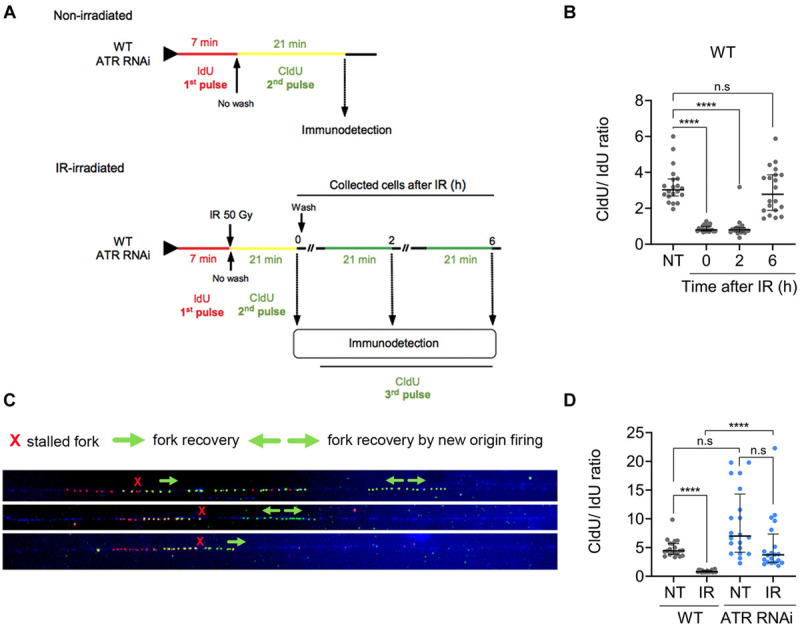
ATR is necessary for replication fork stalling after IR irradiation. **(A)** Scheme shows the DNA combing assay performed with non-irradiated and IR-irradiated WT or ATR RNAi populations. Progressing replication forks were sequentially labeled with asymmetrical pulses of IdU (7 min) and CldU (21 min) in non-irradiated cells or cells pulsed with CldU (21 min) followed by IR irradiation at the preset time intervals. Then, the DNA fork elongation factor (DFEF) was estimated as the ratio between the length of CldU incorporated (yellow track) and the length of IdU incorporated (red track). **(B)** Dot plots representing DFEF calculated for the non-irradiated WT cells and at different time intervals after IR irradiation in single-stranded DNA. The data represent a total of 20 tracks for each case analyzed. **(C)** Representative images of the tracks immunodetected in WT cells 6 h after irradiation showing stalled DNA fork recovery. **(D)** Dot plots representing the calculated DFEF for the WT and ATR RNAi cells before and after IR irradiation. Significant differences are shown as ****P ≤ 0.0001, as determined by Kruskal-Wallis and Dunn’s test for multiple comparisons; n.s., not significant.

In model eukaryotes, many proteins, including components of the replication machinery, are phosphorylated in an ATR-dependent manner in response to IR irradiation (Matsuoka et al., 2007). To investigate whether ATR has a role in replication fork elongation, we performed a DNA combing assay in WT and ATR-RNAi cells. Briefly, progressing replication forks were sequentially labeled with two consecutive asymmetrical pulses of thymidine analogs: the first IdU pulse of 7 min was followed by a second CldU pulse of 21 min in non-irradiated cells or at established times after irradiation with 50 Gy of IR (Figure 5A). Then, the tracks of each of these analogs were immunostained with anti-IdU (red) and anti-CldU (green) antibodies and microscopically visualized (Supplementary Figure S5). In this strategy, we omitted the washing step after the IdU pulse. Thus, after IdU incorporation in the first pulse (red track), CldU incorporation (2nd pulse) occurs simultaneously with the IdU remaining to generate yellow tracts. In this way, ongoing replication forks are visualized as red tracts followed by yellow tracts. On the other hand, we also evaluated the recovery of the stalled replication fork progression after IR treatment. In this case, the cells were washed and cultured in fresh medium after the second CIdU pulse as described above. On the other hand, we also evaluated the recovery of the stalled replication fork progression after IR treatment. In this case, the cells were washed and cultured in fresh medium after the second CIdU pulse as described above. Then, a third pulse of 21 min CldU was added 2 or 6 h after irradiation. Different from the first two-pulse strategy, where the cells are not washed between the first and second pulses, which generates yellow tracks, the third pulse generates green tracks (Figure 5A). From these length tracks, we calculate the DNA fork elongation factor (DFEF), which is the ratio between the length of CldU incorporated after the 2nd pulse (yellow track) or 3rd pulse (green track) and the length of IdU incorporated after the 1st pulse (red track); in a replication fork with regular elongation speed, it is expected to be approximately 3 (21/7).

The DNA-combing analysis showed that in non-irradiated WT cells, the median DFEF obtained was ∼3.027 (interquartile range; IQR of 2.69–3.64), as expected (Figure 5B). In contrast, in irradiated WT cells, we observed a significant decrease in DFEF compared to that in non-irradiated WT cells during the first 2 h (Figure 5B). At 0 and 2 h after irradiation, the median DFEF obtained was 0.8 (IQR of 0.7–1.0 and 0.7–0.9 at 0 and 2 h, respectively), suggesting that the replication fork had stopped during this time. We also investigated whether DNA synthesis was resumed 6 h after irradiation since we had previously observed that repair of IR-induced DSBs in *T. brucei* took ∼6 h (Marin et al., 2018). After this time, DNA synthesis was resumed, showing a median DFEF of 2.78 (IQR: 1.89–3.86), similar to the value found in the non-irradiated WT cells (Figure 5B, 6 h vs. NT). We also observed that DNA synthesis resumption was accompanied by replication events such as unidirectional recovery or new origin firing during the second pulse, which were later detected as green tracks only (Figure 5C).

Next, we investigated the role of ATR in stalling and stabilizing the replication fork before and after DNA damage. For this experiment, ATR-RNAi cells were subjected to a DNA combing assay before and after IR. Before irradiation, the median DFEF obtained was 7.02 (IQR 4.2–14.3) (Figure 5D). After IR irradiation, the median DFEF was 3.7 (IQR: 2.4–7.4), which was similar to the DFEF detected in the non-irradiated WT cells (median: 4.4, IQR 3.8–5.7) (Figure 5D), indicating that the replication fork continued to elongate even in the presence of DNA damage with rate similar to that of the non-irradiated WT cells. Together, these results suggest that ATR is necessary for stalling and stabilizing the replication fork after DNA damage generated by IR irradiation.

### ATR Phosphorylates H2A in a Reduced Percentage of Cells and Is Required for the Relocation and Upregulation of RAD51 Following IR-Induced Damage

Since we observed that ATR is important for proper cell cycle progression of cells under normal culture conditions and for the activation of cell cycle checkpoints in response to IR-induced DSBs, we analyzed whether ATR has a role in the phosphorylation of histone H2A, a DNA damage marker in trypanosomatids, after IR irradiation. In response to DNA damage, histone H2A is phosphorylated on Thr 130, giving rise to γH2A (Glover and Horn, 2012). In unperturbed cells, γH2A is typically detected in a small percentage of cells (∼10%) appearing as discrete nuclear foci (Glover and Horn, 2012). Following exposure to DNA damaging agents, both the percentage of cells and the signal intensity of γH2A can be substantially increased (Glover and Horn, 2012). For example, after methyl methanesulfonate (MMS) treatment, multiple foci were detected (∼50% of cells), and after phleomycin treatment or IR irradiation, γH2A was detected in a dispersed pattern throughout the nucleus in almost all cells (Glover and Horn, 2012; Marin et al., 2018). These differences in detection profiles are clearly related to the type and extent of damage caused by these agents.

**FIGURE 6:**
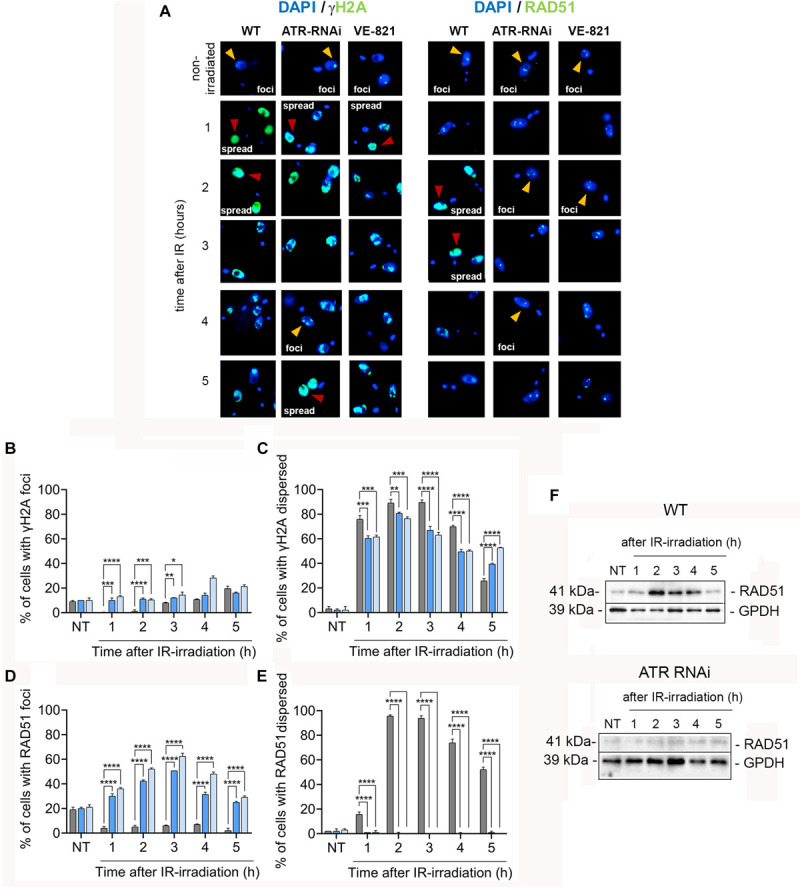
ATR is important for optimizing DNA damage signaling and shows crucial functions in the relocation and upregulation of factors needed for the repair of IR-induced DSBs. **(A)** IFI assay using antisera specific for γH2A and Rad51 proteins involved in DNA damage signaling and repair in WT cells or cells with ATR silenced or ATR inhibited before and after IR irradiation. In each case, the percentage of cells with protein assembly in foci (yellow arrowheads) and the percentage of cells with dispersed staining patterns (red arrowheads) were quantified. Bar plots show the percentage γH2A **(B,C)** and RAD51 **(D,E)** detected before and after irradiation in WT cells and cells with ATR silenced or inhibited. The data represent the averages of three independent experiments, each consisting of n = 150, with error bars representing the standard deviations. Significant differences are shown as ****P ≤ 0.0001, ***P ≤ 0.001, **P ≤ 0.01, and *P ≤ 0.05, as determined by one-way ANOVA and Dunnett’s test for multiple comparisons. **(F)** Western blot analysis of RAD51 protein levels in the WT and ATR RNAi cells.

To investigate whether ATR has a role in the phosphorylation of histone H2A after IR irradiation, we immunodetected γH2A using anti-γH2A antiserum (Figure 6A) and quantified both the percentage of the cells with foci and the percentage of cells with a dispersed staining pattern of γH2A before and after IR irradiation in the WT and ATR silenced or ATR-inhibited cells (Figures 6B,C). Consistent with previous studies, in the unperturbed WT population, ∼10% of the cells carried at least one focus of γH2A, and in < 3% of cells, γH2A was detected as a dispersed staining pattern throughout the nucleus (Figures 6B,C, NT), which may be associated with spontaneous DNA breaks. In the induced or ATR-inhibited population, the percentages of γH2A were similar to those in the WT cells (Figures 6B,C, NT). On the other hand, for 2 h after irradiation, we observed a remarkable increase in the percentage of cells with a dispersed staining pattern, up to ∼90% of the cells, and detection of γH2A foci, in < 1% of the cells, in a WT population (Figures 6B,C, 2 h), which may be explained by generalized DSBs generated by IR irradiation (Marin et al., 2018). From 3 to 5 h postirradiation, the percentage of cells with γH2A dispersed in the nucleus began to decrease, while cells with foci began to increase, both reaching values of ∼20% (Figures 6B,C, 3–5 h), suggesting that the DNA signaling response began to cease, possibly as a result of DNA damage repair, as previously reported (Marin et al., 2018). Similar to WT, both populations (ATRi-induced and ATR-inhibited cells) showed an increased percentage of cells with a dispersed staining pattern of γH2A, reaching maximum values of ∼80% at 2 h and decreasing until reaching ∼ 50% at 4–5 h (Figure 6C, 2 h). However, these percentages were significantly lower compared with the WT population during the first 4 h, and they were higher 5 h after irradiation. Additionally, in these two populations, γH2A was detected in foci in ∼10% of cells even during the first 2 h after irradiation (Figure 6B, 1–2 h). These results indicate that in response to IR-induced DSBs, H2A histone phosphorylation is primarily ATR-independent. However, ATR contributes to the phosphorylation of a small but significant percentage of cells. On the other hand, the constant immunodetection of γH2A up to 5 h compared with that found in the WT population may be explained by the persistent damage as a result of the absence of ATR, which may be required at later stages for efficient DNA repair.

We also determined the γH2A level in WT and ATR inhibited cells after IR irradiation using Western blot analysis. Consistent with immunodetection analysis, the WT population showed an increase of ∼2.3-fold change at first 2 h and ∼2.0-fold change from 3 to 5 h in γH2A level in response to IR irradiation compared to non-irradiated cells, while that ATR inhibited cells showed reduction of ∼30% at fist 2 h and ∼44% from 3 to 5 h (∼1.5 and 1.2-fold change, respectively) in γH2A level compared to irradiated WT population (Supplementary Figures S6A,B,D). Additionally, we determine the γH2A level in ATM inhibited cells after IR irradiation. Similar to ATR inhibited cells, the levels of γH2A in ATM inhibited cells detected were reduced (Supplementary Figures S6C,D). However, different to ATR inhibition, γH2A level in ATM inhibited cells was drastically reduced showing reduction values of ∼81 and 89% at 2 h and from 3 to 5 h, respectively (0.4 and 0.2-fold change related to non-irradiated cells) compared with those in WT population (Supplementary Figures S6A,C,D). These findings indicated that, in response to IR-induced DSBs, the H2A is primarily phosphorylated by ATM kinase. Next, we examined the location of RAD51 recombinase (a key factor involved in late stages of HR-mediated repair) using anti-RAD51 antiserum in an immunofluorescence assay of the three populations: WT, ATR-silenced and ATR inhibited cells (Figure 6A). Similar to γH2A, the RAD51 protein can be detected in formed foci or in a dispersed pattern throughout the nucleus, depending on DNA damage intensity (Hartley and McCulloch, 2008; Marin et al., 2018). Immunofluorescence analysis using anti-RAD51 serum showed that, in the three non-irradiated populations (WT, ATR-silenced and ATR-inhibited cells), at least one RAD51 focus was found in ∼20% of the cells, and a dispersed staining pattern of RAD51 was found in ∼3% of the cells (Figures 6D,E). After IR irradiation and during the first 5 h, we detected a lower percentage of cells with RAD51 foci (<10%), which was accompanied by an increase in the percentage of cells with a dispersed staining pattern of RAD51 in the WT population (Figures 6D,E). Thus, 1 h after irradiation, ∼16% of the cells had a dispersed staining pattern of RAD51; at 2–3 h, the percentage of cells with this pattern increased, reaching values of ∼95%, and at 5 h, the percentage of cells with this pattern decreased by 52% (Figure 6E). In contrast to WT cells, the increase in the detection of RAD51 in the ATR- silenced or ATR-inhibited population after irradiation was dependent on the number of cells with foci and not on the number of cells with a dispersed staining pattern for this protein (Figures 6D,E). Thus, we observed an increase in the percentage of cells with RAD51 foci by as much as ∼30% at 1 h, detected maximum values of ∼60% at 3 h and finally observed a decrease to ∼29% at 5 h (Figure 6F). Thus, these results indicate that ATR is required for the proper recruitment of RAD51 to break sites.

It was recently reported that ATR plays a critical role in maintaining protein levels that are essential for HR-mediated repair (Kim et al., 2018). Considering this finding, we asked whether ATR might also be involved in modulating RAD51 expression levels in response to damage caused by IR. To answer this question, we performed a Western blot analysis and compared RAD51 expression levels in the ATR-silenced population with those in the WT population after IR irradiation. Consistent with the patterns observed in the immunodetection assay, we found an increase in RAD51 expression levels in response to irradiation during the first 4 h in the WT population (Figure 6F, top). However, in the ATR-silenced population, we did not observe any increase in the expression levels of RAD51 in response to DNA damage at the same evaluation times (Figure 6F, bottom). Together, these results suggest that ATR is important not only for the relocation but also for the upregulation of the RAD51 protein in response to IR-induced DSBs.

## Discussion

We investigated the role of *T. brucei* ATR in response to IR-induced DSBs and showed that this kinase plays essential functions in the control of several processes of DDR together with cell cycle coordination by checkpoint activation. ATR is required for proper cell cycle progression and is involved in intra-S checkpoint activation with some contribution in G1/S checkpoint modulation in response to IR-induced DSBs. Moreover, we found that, after irradiation, ATR is required for stalling the replication fork, is involved in the regulation of DNA damage through H2A histone phosphorylation (γH2A) and is necessary for the recruitment and expression upregulation of critical factors for HR, such as RAD51. Together, these results suggest that procyclic *T. brucei* ATR acts as an apical kinase to coordinate the DNA damage response to IR-induced DSBs.

By employing a dual-pulse sequential labeling strategy with two thymidine analogs to monitor cell progression through the S phase (Figures 1A–D), we found that ATR is necessary for proper progression through the S phase under normal culture conditions. This finding was demonstrated by the increase in the percentage of intra-S cells in the ATR-silenced population subjected to dual labeling with thymidine analogs (Figures 2D,E). This finding suggests that *T. brucei* ATR may have critical functions similar to those described in other organisms, where this kinase is activated in S phase, presumably to repair damaged replication forks, regulate replication origin firing and avoid premature entry into mitosis (Cimprich and Cortez, 2008). On the other hand, our results also indicate that intra-S checkpoint activation in response to IR-induced DSBs is mainly controlled by ATR. We also observed that IR irradiation of *T. brucei* WT cells triggered a strong intra-S checkpoint, as determined by the dual labeling strategy with IdU and CldU (Figure 3A). Additionally, cells accumulated in G1 after IR irradiation during the evaluated time (Figure 4B), similar to the findings reported for *Leishmania major*, but differently from those reported for *Trypanosoma cruzi*, where cells accumulated predominantly in the G2/M phase after IR irradiation (Garcia et al., 2016). However, under conditions of ATR silencing or inhibition, the cells that were in S phase during IR irradiation continued to progress through S phase, similar to the WT non-IR-irradiated cells, suggesting that intra-S checkpoint activation is mainly mediated by ATR (Figures 3B,C). In model eukaryotes, ATR plays an important role not only in controlling the intra-S-phase checkpoint during normal S-phase progression but also in responding to DNA damage mainly induced by replication stress (Cimprich and Cortez, 2008; Saldivar et al., 2017). However, in cells with IR-induced DSBs, the intra-S checkpoint is primarily controlled by ATM since this kinase is quickly recruited to and activated at break sites (Paull and Lee, 2005; Shiloh and Ziv, 2013), in contrast to ATR, which is indirectly activated by ssDNAs generated from DSB resection, a process promoted by ATM (Adams et al., 2006; Cuadrado et al., 2006; Jazayeri et al., 2006). Our results showed that *T. brucei* ATR seems to play a more prominent role in the activation of the intra-S checkpoint followed by IR irradiation since we observed a complete abrogation of this checkpoint in ATR-silenced and ATR-inhibited cells under the evaluated conditions. How *T. brucei* ATR modulates intra-S checkpoint activation, however, is a question that requires further study. In addition to intra-S checkpoint control, we observed that *T. brucei* ATR can partially modulate G1/S checkpoint activation after IR irradiation. This was indicated by the early detection of new cells entering the S phase after IR from the ATR-silenced population subjected to a dual-labeling pulse (Figure 3B). In model eukaryotes, it is widely accepted that G1/S checkpoint activation is mainly controlled by ATM, whereas the activation of the intra-S phase and G2/M checkpoints are regarded as ATR functions. This supposition is corroborated by the fact that the DSBs in G1 are not resected to generate significant amounts of RPA-ssDNA to activate ATR (Jazayeri et al., 2006). However, recent studies in human cells have shown that ATR can be activated in the G1 phase in response to IR irradiation, indicating that its activation does not require extensive DNA end resection as previously suggested (Gamper et al., 2013). This new evidence is consistent with our findings, which support a possible role for *T. brucei* ATR in G1/S checkpoint control. An important question arises from this scenario: Why does a microorganism that apparently lacks a canonical NHEJ repair pathway maintain a G1 checkpoint? One possibility is that the DSBs generated in G1 may be repaired in this phase. Considering that HR and MMEJ are the two predominant DSB repair mechanisms in *T. brucei* and that HR is restricted to the S and G2 phases, the DSBs in G1 could be repaired by MMEJ, since this mechanism is also active in G1, as reported in human cells (Xiong et al., 2015). However, additional studies will be necessary to determine the repair mechanism used in this phase of the cell cycle in *T. brucei.*

We also found that the procyclic *T. brucei* ATR kinase is required for stalling the replication fork after DNA damage caused by IR. In non-irradiated WT and ATR-silenced *T. brucei* cells, the replication fork elongation process was similar, with greater variation in the ATR-silenced population than in the WT population (Figure 5D). However, we observed that, after IR irradiation, the ongoing replication forks stalled in *T. brucei* WT cells, while in the ATR-silenced population, the replication forks continued to elongate at rates similar to those of the non-irradiated WT cells (Figure 5D), indicating that ATR plays an important role in the modulation of fork speed in response to DNA damage induced by IR. In agreement with our results, it has been demonstrated in humans that ATR can control replication fork stability through several processes. For example, ATR can regulate fork reversal via the phosphorylation of SWI/SNF-related matrix-associated actin-dependent regulator of chromatin subfamily A-like protein 1 (SMARCAL1) (Couch et al., 2013), prevent RPA exhaustion through suppression of late-origin firing (Toledo et al., 2013), and regulate dNTP availability (Buisson et al., 2015). However, despite the evidence, the molecular mechanisms by which ATR regulates the stability of the replicating fork remain to be determined. Additionally, most previous studies were performed under replication stress conditions, in which ATR is quickly activated by RPA-ssDNA. Thus, little is known about the role of ATR in replication fork stability in the context of IR-induced DSBs; according to the available information, ATR activation likely occurs after ATM activation (Adams et al., 2006; Cuadrado et al., 2006; Jazayeri et al., 2006), resulting in a particularly complex scenario. On the other hand, the treatment of cells with irradiation led to recovery of fragile and breakable DNA fibers mainly in ATR-RNAi cells, so we could only analyze a limited number of molecules. Finally, further investigations will be required to mechanistically determine how *T. brucei* ATR controls replication fork stalling in response to IR-induced damage.

Our data indicated that, although histone H2A is not primarily phosphorylated by ATR in response to IR-induced DSB, ATR contributes to H2A phosphorylation in a small but significant percentage of cells. In model eukaryotes, H2AX is a critical player in the DDR, and once phosphorylated, it creates a zone around a DSB site, facilitating the recruitment of proteins that participate in signaling, DNA repair and cell cycle checkpoint activation (Celeste et al., 2002). In *T. brucei*, the equivalent of γH2AX is phosphorylated histone H2A at Thr130, which forms γH2A (Glover and Horn, 2012). In our results we observed that, in response to IR-induced DNA damage, procyclic *T. brucei* WT cells triggered robust phosphorylation of histone H2A (Figures 6B,C). In contrast, the cells with silenced or inhibited ATR showed a γH2A dispersed staining pattern during the first 2 h which subsequently decreased from 3 to 5 h (Figures 6B,C) and moderated reduction of γH2A protein level (Supplementary Figures S6B,D). On the other hand, the ATM inhibition show led to a drastic reduction of γH2A protein level after irradiation (Supplementary Figures S6C,D). This indicates that ATR is not critical for IR-induced γH2A formation, but it has a complementary role in IR-dependent H2A phosphorylation. In human cells, H2AX is mainly phosphorylated by ATM after low doses, while at higher doses of IR irradiation, other kinases, such as ATR or DNA-dependent protein kinase (DNA-PK) (latter is also stimulated by DSBs and involved in NHEJ-mediated repair), can contribute to H2AX phosphorylation (Burma et al., 2001; Stiff et al., 2004). These observations may be related to the specific functions of each kinase. Thus, ATM is quickly activated in response to DSBs, showing a predominant role in the initial steps of signaling and repair, while ATR activation, in this context, is delayed, with a major role in later steps of DNA repair. Considering that an ATM homolog has been previously identified in *T. brucei* (Genois et al., 2014), and it differs from DNA-PK kinase, which seems to be absent or divergent in this microorganism, the possible candidate for IR-dependent H2A phosphorylation in absence of ATR inhibition could be ATM kinase. However, this hypothesis needs to be validated. On the other hand, the higher levels of γH2A detected at 5 h in the population with silenced or inhibited for ATR compared to those of with WT population (Figure 6C) can be attributed to the unrepaired DNA damage that persisted as a result of the absence of a related ATR function. This reasoning is supported by two facts observed in other organisms: first, the clearance of γH2AX at DSB sites is generally related to the completion of DNA repair at these break sites (Bouquet et al., 2006), and second, there is evidence showing that ATR knockdown inhibits the clearance of γH2AX foci, while its overexpression leads to rapid attenuation of increased γH2AX foci, in relation to control cells, after IR exposure (Kim et al., 2011). In agreement with these pieces of evidence, our results suggest that procyclic *T. brucei* ATR may have a more important role in the later stages than in the initial stages of IR-induced damage signaling, while other kinases related to ATR may be critical for this function.

Our results also show that ATR participates in direct or indirect recruitment and is required for the upregulation of the RAD51 expression levels following IR irradiation. In a previous report, we showed that IR-induced DSBs can activate an efficient DNA damage response (DDR), recruiting key factors for HR-mediated repair at the late S/G2 phases in the insect stage of *T. brucei* (Marin et al., 2018). In agreement with this study, we observed that 2 h after IR irradiation, RAD51 quickly relocated to break sites, as demonstrated by the dispersed staining pattern of RAD51 throughout the nucleus in most cells (90%) in the WT population (Figure 6E). Within 3-5 h post irradiation, the dispersed staining pattern was slowly lost after being detected in 52% of the cells, indicating an ongoing DSB repair process (Figure 6E). In contrast, in the ATR-silenced and ATR-inhibited population, we observed an increase in the percentage of cells with foci (∼60% at 3 h and decreasing until ∼29% at 5 h), but we did not observe an increase in the percentage of cells with a RAD51 dispersed staining pattern as in the case of the WT population after IR irradiation (Figures 6D,E). In addition to the impaired recruitment of RAD51, these cells did not show an upregulation of RAD51 expression in response to irradiation, as observed in the WT population (Figure 6F). These results indicate that ATR is required for the proper recruitment and upregulation of RAD51 expression levels after DNA damage caused by IR. The formation of the RAD51 dispersed staining pattern might be associated with the amount of DNA damage, which is preceded by an increase in the number of RAD51 foci in response to this damage. Thus, in exacerbated DNA damage, RAD51 foci may no longer be viewed as separate units, and instead, the RAD51 recruited at multiple DNA damage sites may be detected as a dispersed staining pattern. In line with this supposition, a possible explanation for the increase in the percentage of cells with foci in the ATR-silenced population is that the formation of these foci may be related to residual ATR activity since the silencing of this kinase in cells with an inducible RNAi system did not reach 100% in 48 h. A similar explanation may apply to ATR-inhibited cells since we have no verifiable means to assess the activity of inhibitors, such as CHK1 phosphorylation. If this supposition accurately depicts the situation, residual ATR activity may be promoting the limited recruitment of RAD51 to sites of damage; therefore, it is possible to observe the formation of some cells with foci. As the optimal response to damage requires RAD51 upregulation and ATR-silenced ATR-inhibited cells cannot induce its upregulation, the absence of RAD51 upregulation can be the cause of the absence of the RAD51 dispersed pattern observed in the WT population. In agreement with our findings, there is a growing number of studies showing that ATR is involved in the regulation of essential factors for HR-mediated repair. It has been frequently observed that IR-irradiated human cells previously treated with ATR inhibitors show a remarkable reduction in RAD51 foci formation (Buisson et al., 2017). Additionally, it was demonstrated that ATR enhances the BRCA1-PALB2 interaction through the phosphorylation of PALB2 at S59 after IR irradiation, a critical step that promotes RAD51 filament formation (Buisson et al., 2017). More recently, it was demonstrated that ATR-CHK1 signaling is required for ensuring the proper expression of key components of the HR machinery, such as RAD51, which directly affects the ability of cells to undergo HR-mediated repair (Kim et al., 2018). Consistent with these studies, our preliminary results show that *T. brucei* ATR is also a key factor in recruiting and regulating essential proteins for HR-mediated repair. We do not know whether RAD51 is recruited directly or indirectly by ATR in *T. brucei*. Similarly, we do not know whether *T. brucei* ATR regulates the abundance of factors through transcription, as reported in human cells. Another possibility that may explain the alteration of the appropriate recruitment and abundance of recombination factors is that checkpoint inactivation does not ensure sufficient time to recruit the factors required for DNA damage repair, since the function of the checkpoint is to arrest the cell cycle until the damage is repaired.

In summary, our findings suggest that ATR has an important role in regulating the DDR of IR-induced DSBs in the PCF of *T. brucei* to guarantee their survival through controlled and efficient DNA repair. Additionally, the understanding of how the parasite addresses DNA damage may be helpful for the development of potential therapies focused on parasite-specific genomic and molecular processes for the treatment of HAT.

## Materials and Methods

### Cell Culture, Transgenics, and Ionizing Radiation (IR) Treatment

Procyclic forms (PCFs) of *Trypanosoma brucei* (Lister strain 427) were cultured at 28°C in SDM79 medium supplemented with 10% (v/v) fetal bovine serum. To analyze strains with ATR inhibited by RNAi, we used pQ117-PCF cells. pQ117-PCF cells were engineered procyclic forms of strain 29.13 showing resistance to G418, hygromycin, and phleomycin and expressing a tetracycline regulatable RNA interreference (RNAi) construct targeting the ATR gene. Briefly, to generate these cells, a 425 bp fragment of the Tb927.11.14680 gene (nt 4,242–4,666) was selected using RNAit software (Redmond et al., 2003) and amplified by PCR with primers containing designed *Bst*XI sites (forward: 5′-ATACCAATGTGATGGCGCTCCCTTAAGTGCAAAAG-3′; and reverse: 5′-ATACCATAGAGTTGGCGAATTCCCTCCAA TGAAGA-3′), as previously described (Inoue et al., 2005). Ligation of the fragment into a *Bst*XI-digested pQuadra3 vector (Inoue et al., 2005) generated the pQ117 vector, which contains inverted 425 bp repeats of the gene separated by spacer regions. *Not*I-digested pQ117 was used to transfect PCF cells from strain 29.13 and were selected based on their resistance to phleomycin, as previously described (Inoue et al., 2005). The pQ117-PCF cells carry the pQ117 vector integrated into the silent rDNA spacer for tetracycline-inducible RNA interference (RNAi); this vector confers resistance to phleomycin. In vector nomenclature, “117” refers to the gene Tb927.11.14680 (annotated as phosphatidylinositol 3-related kinases, a putative ATR). These cells were named *T. brucei 477* PCFs during the engineering process. In general, during IR treatment, exponentially growing parasites (∼3–10 × 10^6^ cells/mL) from each of strain were subjected to 50 Gy from a Gamma Cell 220 cobalt 60 irradiator unit with a rate dose of 913 Gy/h.

### Kinase Inhibitors and Cell Viability Assay

Exponentially growing parasites were subjected to different concentrations of ATR kinase inhibitor (VE-821, from Sigma Aldrich) or ATM kinase inhibitor (KU55933, from Sigma Aldrich) to determine the optimal concentration of each kinase inhibitor, as indicated by its failure to impair long-term cell viability, by dose-response curves. The cell density after kinase inhibitor exposure was determined daily for 5 days in a Z Series Coulter Counter set for 5–15 μM (counting parameters). Complete culture medium and the kinase inhibitor were refreshed every 2 days, and the parasites were maintained at 28°C.

### Dual-Pulse Sequential Labeling of DNA With Two Thymidine Analogs, IdU and CldU

Exponentially growing *T. brucei* strain 427 PCF (WT) or *T. brucei* strain 477 PCF, which was tetracycline induced for 48 h (ATR-RNAi), were incubated in the presence of IdU (100 μM) for 30 min. At the end of the IdU pulse, the WT parasites were submitted to different conditions: non-treated (control), 50 Gy of IR (IR-irradiated), VE-821 (5 μM) + 50 Gy or KU55933 (20 μM) + 50 Gy, while the ATR-RNAi strain was untreated (ATR-RNAi non-irradiated) or subjected to ATR-RNAi + 50 Gy of IR (ATR-RNAi IR-irradiated). Afterward treatment, each culture was centrifuged at 1,700 *g* for 5 min to remove the thymidine analogs, the parasites were resuspended in complete culture medium and the kinase inhibitor was added as previously described. To evaluate the G1/S transition or intra-S progression, samples were collected hourly for 5 h, and a second thymidine analog pulse (using 100 μM CldU) was carried out 30 min before collection. Then, the collected parasites were washed twice with 1x PBS, fixed for 15 min with 300 μL of 4% paraformaldehyde, and washed again with 1x PBS. Next, the parasites were scattered onto poly-L-lysine-coated slides, permeabilized with 0.2% Triton X-100 (diluted in 1x PBS) for 15 min and washed twice with 1x PBS. Then, the parasites were treated with HCl 2.5 M for 20 min at room temperature, neutralized with 0.2 M borate buffer for 10 min and washed twice with 1x PBS. Then, the parasites were incubated in blocking solution (0.1% Triton X-100, 1% BSA in 1x PBS) for 20 min at room temperature and then incubated for 1 h with the specific anti-IdU antibody [Anti-BrdU (mouse), ref: 347580, Becton Dickinson] diluted 1: 300 in blocking solution. After two washes using 1x PBS + 0.05% Tween 20, the parasites were incubated for more 1 h with specific anti-CldU antibody [Anti-BrdU (rat) ref: OBT0030-BU1/75, ACCU-SPECS] diluted 1:300 in blocking solution. After two washes with 1x PBS + 0.05% Tween 20, the parasites were blocked with 50% FBS (diluted in 1x PBS) for 30 min and incubated with secondary antibodies: Alexa Fluor 568-conjugated anti-mouse and Alexa Fluor 488-conjugated anti-rat antibodies, each diluted 1:500 in blocking solution for 1 h. Finally, the parasites attached to the slides were washed twice and sealed using VECTASHIELD^®^ antifade mounting medium containing DAPI. The images were acquired using an Olympus Bx51 fluorescence microscope (100x oil objective) attached to an EXFO Xcite series 120Q lamp and a digital Olympus XM10 camera controlled by Olympus Cell F software. Image capture conditions were set using unlabeled cells as references.

### DNA Combing Assay

Exponentially growing parasites of both strains [the *T. brucei* 427 PCF strain (W.T.) or *T. brucei* pQ117-PCF strain (tetracycline induced) for 48 h (ATR-RNAi)] were incubated in the presence of IdU (100 μM) for 7 min. Immediately after the parasites were irradiated with 50 Gy, a second thymidine analog pulse (100 μM CldU) was performed for 21 min, without washing the cells between the two pulses. To evaluate the recovery of the stalled replication fork progression after IR treatment, the parasites were centrifuged at 1,700 g for 5 min, washed with 1x PBS and resuspended in complete culture medium. In this case, a third pulse was performed using 100 μM CldU for 21 min 2 or 6 h after IR-irradiation. Then, the parasites were washed twice with 1x PBS + 10 mM glucose and resuspended in 100 μL of 1% low-melting agarose diluted in 1x LB buffer (0.1 M EDTA at pH 8.0, 10 mM Tris-HCl, and 20 mM NaCl). After solidification, the plugs were placed in 300 μL of lysis buffer (0.5 M EDTA at pH 8.0, 1% N-lauroylsarcosine sodium salt, and 100 μg/mL proteinase-K) at 50°C for 24 h. The next day, the plugs were resuspended in fresh lysis buffer for another 24 h. On the third day, the plugs were rinsed with 0.5 M EDTA, pH 8.0, to remove excess lysis buffer. Then, the plugs were washed with T_1__0_E_1_ solution (10 mM Tris-HCl and 1 mM EDTA, pH 8.0) hourly for 3 h. For the last wash, the plugs were maintained in a T_1__0_E_1_ solution overnight at 4°C protected from light. The next day, the plugs were incubated in 1 mL of 0.5 M MES buffer, pH 5.5, at 68°C for 20 min and then at 42°C for 10 min. Next, 2 μL of β-agarose enzyme (EO0461, Thermo Scientific) was added for each plug, and the tubes were maintained overnight at 42°C. The next day, 1 mL of MES buffer, pH 5.5, was added to the reservoirs of a FiberComb machine (Genomic Vision). The digested plugs were carefully tipped into the reservoirs to be stretched onto a coverslip. Then, the coverslips containing stretched DNA were incubated at 65°C for 4 h protected from light. After fixation, the DNA was denatured using a solution of 0.5 M NaOH and 1 M NaCl for 8 min at room temperature and neutralized by washing (twice) with 1x PBS for 3 min each. Next, the DNA on the coverslips was dehydrated using different concentrations of ethanol: 70, 90, and 100% for 3 min/each treatment. The coverslips were air-dried and then blocked with a solution containing 1x PBS, 1% BSA, and 0.1% Triton X-100 at 37°C for 30 min. After blocking, the coverslips were incubated with 20 μL of a solution containing 4 μL of primary antibodies: 3 μL anti-IdU antibody (Anti-BrdU ref: 347580, Becton Dickinson) and 1 μL of anti-CldU (Anti-BrdU ref: OBT0030 -BU1/75, ACCU-SPECS) in 3% BSA diluted in 1x PBS at 37°C for 1 h. After washing (1x PBS + 0.05% Tween 20), the coverslips containing the DNA were incubated in 20 μL of a solution containing the secondary antibodies: 2 μL of Alexa Fluor 568-conjugated anti-mouse and 2 μL of Alexa Fluor 488-conjugated anti-rat diluted in 3% BSA in 1x PBS, at 37°C, for 45 min. After washing, the coverslips were incubated in 20 μL of an antibody solution containing 1 μL of primary anti-single-strand DNA antibody (MAB3868, Millipore Corp.) diluted in 3% BSA in 1x PBS at 37°C for 1 h. After washing, the coverslips were incubated with 20 μL of a solution containing 3 μL of Alexa Fluor 350-conjugated anti-mouse secondary antibody diluted in 3% BSA in 1x PBS at 37°C for 45 min. After washing, the coverslips were sealed onto slides with 5 μL of Prolong^®^ Gold antifade mounting reagent. The slides were then analyzed using an Olympus BX51 fluorescence microscope with an Olympus XM10 digital camera controlled by Olympus Cell F software.

### Immunofluorescence Assay (IFA)

Parasite samples under the different analysis conditions were harvested by centrifugation at 1,700 *g* for 5 min and washed twice with 1x PBS. Then, the parasites were fixed for 15 min using 4% paraformaldehyde with gentle agitation. Next, the parasites were washed, homogenized in 1x PBS, and allowed to adhere onto Teflon-coated slides (Tekdon) for 15 min. Then, the parasites were washed three times (2 min each time) with blocking solution (1x PBS + 3% BSA), permeabilized for 10 min with 0.1% Triton X-100 diluted in 1x PBS and washed three more times. Then, the parasites were incubated at room temperature for 2 h with different antisera according to the analysis: anti-γH2A (Glover and Horn, 2012) or anti-RAD51 (Proudfoot and McCulloch, 2005) antibody (both kindly provided by Dr. Richard McCulloch, University of Glasgow). All antisera used were diluted to 1:1,000 in 1% BSA in 1x PBS. Next, the parasites were washed three times and incubated with blocking solution for 20 min. Then, the parasites were incubated for 1 h with Alexa Fluor 488-conjugated anti-rabbit secondary antibody (Thermo Scientific) diluted at 1:500 in 1x PBS with 1% BSA. After washing, the slides were sealed using 2 μL VECTASHIELD^®^ antifade mounting medium containing DAPI per well. The images were acquired using an Olympus Bx51 fluorescence microscope (100x oil objective) attached to an EXFO Xcite series 120Q lamp and a digital Olympus XM10 camera controlled by Olympus Cell F software. Image capture conditions were set using unlabeled cells as references.

### Western Blotting

Parasite samples under different analysis conditions were harvested by centrifugation at 1,700 *g* for 5 min and washed twice in 1x PBS. Samples were then prepared for total protein extraction in 2x reducing sample buffer containing 1 M Tris-HCl, pH 7.0; 20% SDS; 5% glycerol; 0.1% bromophenol blue; and 5% β-mercaptoethanol. The samples were then boiled for five min at 95°C, separated by SDS-PAGE (30 μL of protein sample per lane) and transferred electrophoretically to nitrocellulose membranes (GE Life Science). After blocking overnight with 1x Tris-buffered saline (1x TBS) containing 5% non-fat dry milk, the membranes were washed with 1x TBS with 0.05% Tween 20 five times for five min each time. After washing, the membranes were cropped and incubated at room temperature under gentle agitation for 4 h with the respective antiserum solution in 1x TBS with 3% non-fat dried milk containing anti-Rad51 (Proudfoot and McCulloch, 2005) diluted 1:500, anti-γH2A antibody (Glover and Horn, 2012) diluted 1:5,000 or anti-GAPDH antibody diluted 1:5,000 used as a loading control (kindly provided by the Laboratory of Biochemistry of Tryps, LaBTryps). After washing, the blots were incubated with horseradish peroxidase (HRP)-conjugated secondary antibody diluted 1:3,000 for 1 h at room temperature. Following additional washes, antibody binding was detected with an Immobilon Western Chemiluminescent HRP substrate (Millipore). Digital images of the membranes were acquired using a UVITEC chemiluminescence and fluorescence imaging system (UVITEC Cambridge).

### Total RNA Extraction

*T. brucei 477* PCFs were maintained in complete SDM79 culture medium containing 2.5 μg/mL phleomycin, 15 μg/mL G418, and 25 μg/mL hygromycin B at 28°C. For total RNA quantification, the parasites were treated with tetracycline 1 μg/mL for 48 h. Approximately 5 × 10^7^ parasites were harvested at 12, 24, and 48 h after centrifugation at 1,700 *g* for 5 min. Then, the pellets were homogenized in 750 μL of TRIzol by gentle pipetting. Then, 200 μL of chloroform was added, and the samples were homogenized by inversion and incubated at room temperature. To allow the separation of the phases, the samples were centrifuged at 12,000 *g* for 15 min at 4°C. The aqueous phase was placed in a new tube, and 500 μL of 100% isopropanol was added for homogenization by gentle inversion for 10 min. To obtain total RNA, the pellets were washed with fresh 75% ethanol in 0.1% DEPC water and centrifuged at 7,500 *g* for 5 min at 4°C and air-dried for 15 min. The pellets were resuspended in 20 μL of DEPC water and incubated in a heat block at 55°C for 10 min. Total RNA samples were quantified using a NanoDrop 2000C and finally stored at −70°C for RT-qPCR quantification.

### Real-Time RT-qPCR

The oligonucleotides qPCR117-F (5′-TGATGGTATTCTGT GCCGTT-3′) and qPCR117-R (5′-CTGCCCAGTGAATCTGCT TA-3′) were used to verify the knockdown of the Tb927.11.14680 gene (phosphatidylinositol 3-related kinases, the putative ATR). The primers were diluted at 100 μM in sterile water and stored at −20°C. The SuperScript^TM^ III kit was used for cDNA synthesis. In brief, 14 μL of a solution containing 4 μg of RNA, 1 μL of oligo dT and 1 μL of dNTP mix (10 mM) was incubated at 65°C for 5 min. Then, 4 μL of 5x First Strand buffer, 1 μL of DTT (0.1 M) and 1 μL of reverse transcriptase to a final volume of 20 μL was used for cDNA synthesis. Tubes were incubated at 50°C for 1 h, and inactivation was performed at 70°C for 15 min. For RT-qPCR, a solution containing 2.5 μL of qPCR117-F 2.4 μM) and qPCR117-R (2.4 μM) oligonucleotides, 10 μL PowerUp^TM^ SYBR^®^ Green Master Mix and 5 μL of cDNA (8 ng/μL) was used. Quantification was performed in the StepOnePlus thermocycler real-time PCR system according to the following program. Step 1: (1x) at 95°C for 10 min; step 2: (40x) at 95°C/15 s + 60°C/1 min; melting curve: (1x) at 95°C/15 s + 60°C/1 min + 95°C/15 s. the Data were exported from the apparatus, and the threshold cycle (CT) was obtained with the LinRegPCR computer program. The relative quantification of the ATR gene was performed using the Schmittgen method with the equation 2^–^*^ΔΔ*Ct*^.*

### Statistical Analysis

All graphic representations were generated and statistical analyses were performed based on a minimum of three independent experiments with GraphPad Prism software (version 8.0). The tests used and significant differences are shown in the corresponding figure legends.

## Data Availability Statement

The original contributions presented in the study are included in the article/Supplementary Material, further inquiries can be directed to the corresponding author/s.

## Author Contributions

ME supervised and designed the study. CM and IC participated to the experimental design. PM, RO, RP, MS, CBA, AL, and CA performed the experiments. ME, RO, RP, and PM analyzed and interpreted the data. ME, RO, RP, and PM wrote the manuscript. IC contributed to new reagents and analytic tools. All authors contributed to the article and approved the submitted version.

## Conflict of Interest

The authors declare that the research was conducted in the absence of any commercial or financial relationships that could be construed as a potential conflict of interest.
