# Supplementary material for: ATR Kinase Is a Crucial Player Mediating the DNA Damage Response in Trypanosoma brucei
Source: Front Cell Dev Biol. 2020 Dec 22;8:602956. doi: 10.3389/fcell.2020.602956 (PMC7783291; doi:10.3389/fcell.2020.602956)
Supplement: Supplementary file 1 [file Data_Sheet_1.PDF]

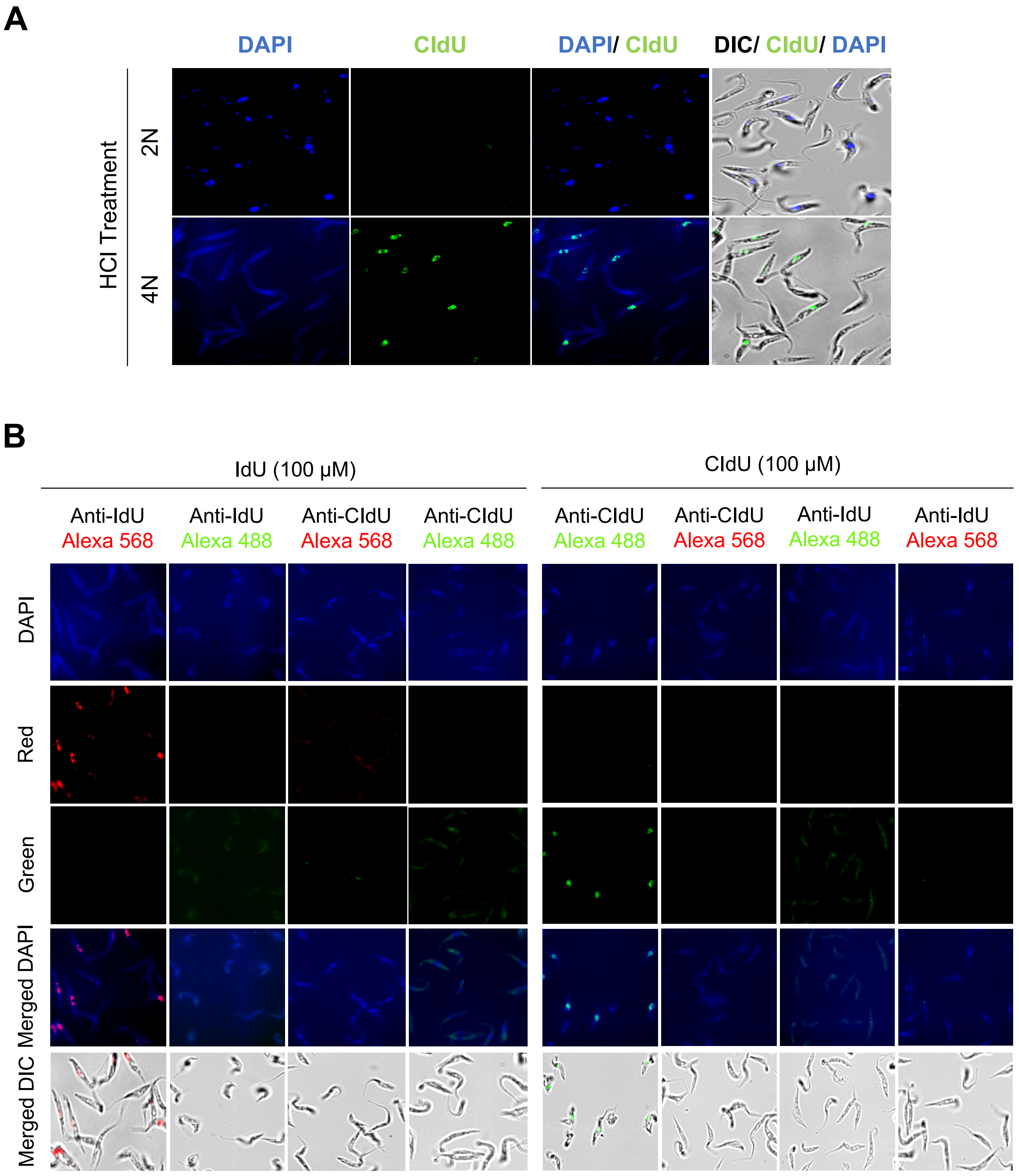

Supplementary Figure S1. The codetection of thymidine analogs is specific. (A) Procyclic forms of *T. brucei* 427 were treated with HCl 2N or 4N for the detection of incorporated CldU analogs. **(B)** Secondary antibody-specific test: as demonstrated in the figure, there was no cross detection among the antibodies used in the experiment. The secondary antibody for one thymidine analog does not detect the primary antibody for the other analog. From top to bottom: DAPI staining; IdU-incorporated detection using anti-IdU and processed for detection using the respective secondary antibody (Alexa Fluor 568); CldU-incorporated detection using anti-CldU and processed for detection using the respective secondary antibody (Alexa Fluor 488).

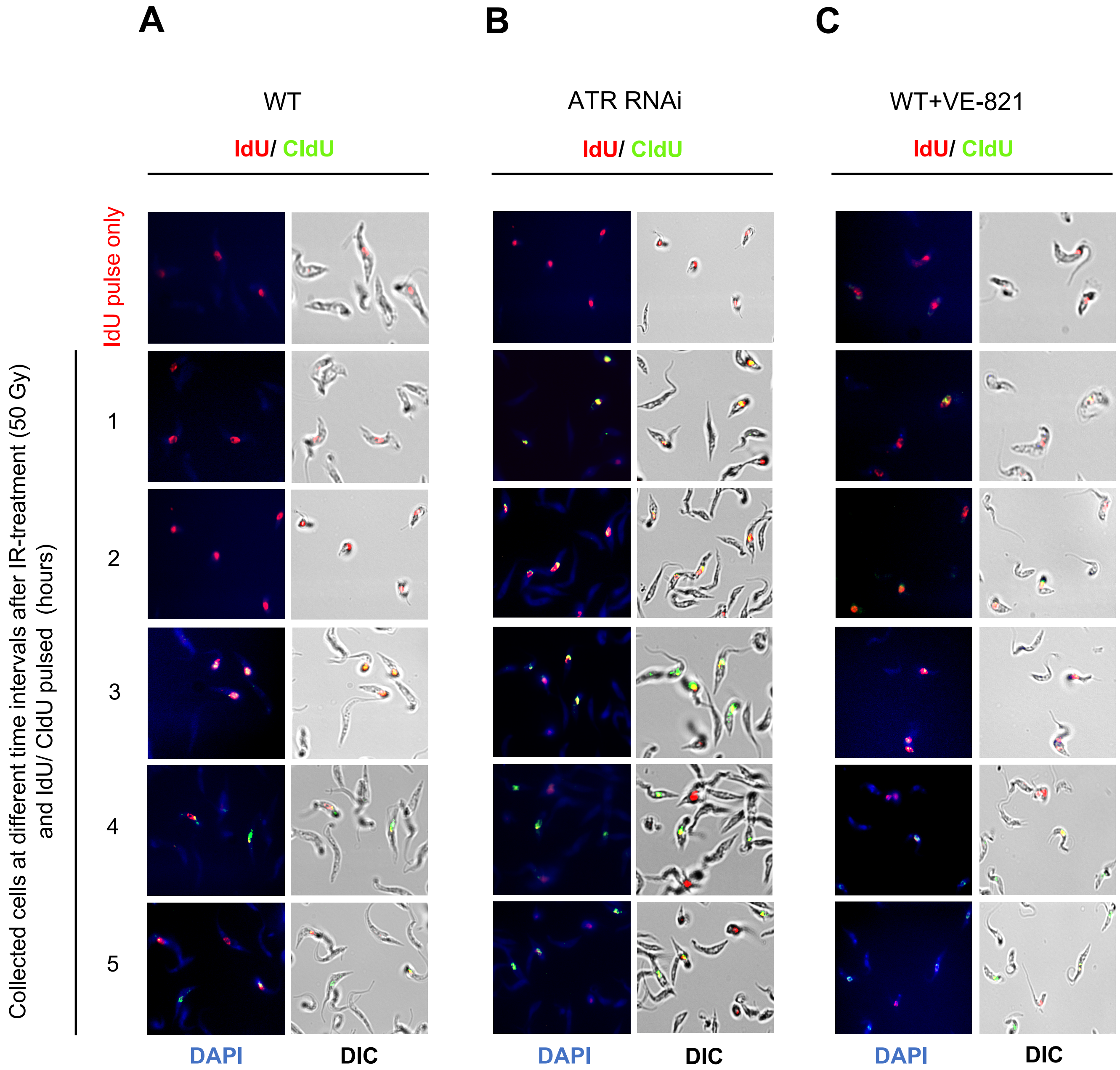

Supplementary Figure S2. Codetection of thymidine analogs followed by IR irradiation. (A-C) Immunodetection of thymidine analogs incorporating IdU (red), CldU (green) and both analogs IdU/CldU (yellow) in the WT, ATR-silenced and ATR-inhibited populations after irradiation with 50 Gy of IR.

A

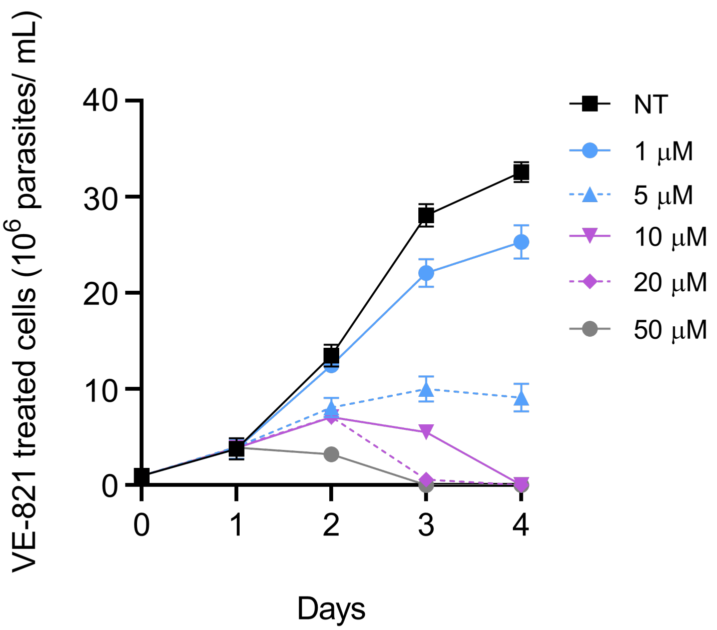

Supplementary Figure S3. Cellular viability of *T. brucei* PCFs after treatment with an ATR kinase inhibitor. (A) Graph shows cell growth of the procyclic forms of *T. brucei* after exposure to the ATR inhibitor. Different concentrations of the ATR kinase inhibitor VE-821 were used (1-50 μM). The data represent the average of three independent experiments, and the bars represent the standard deviations. NT, not treated.

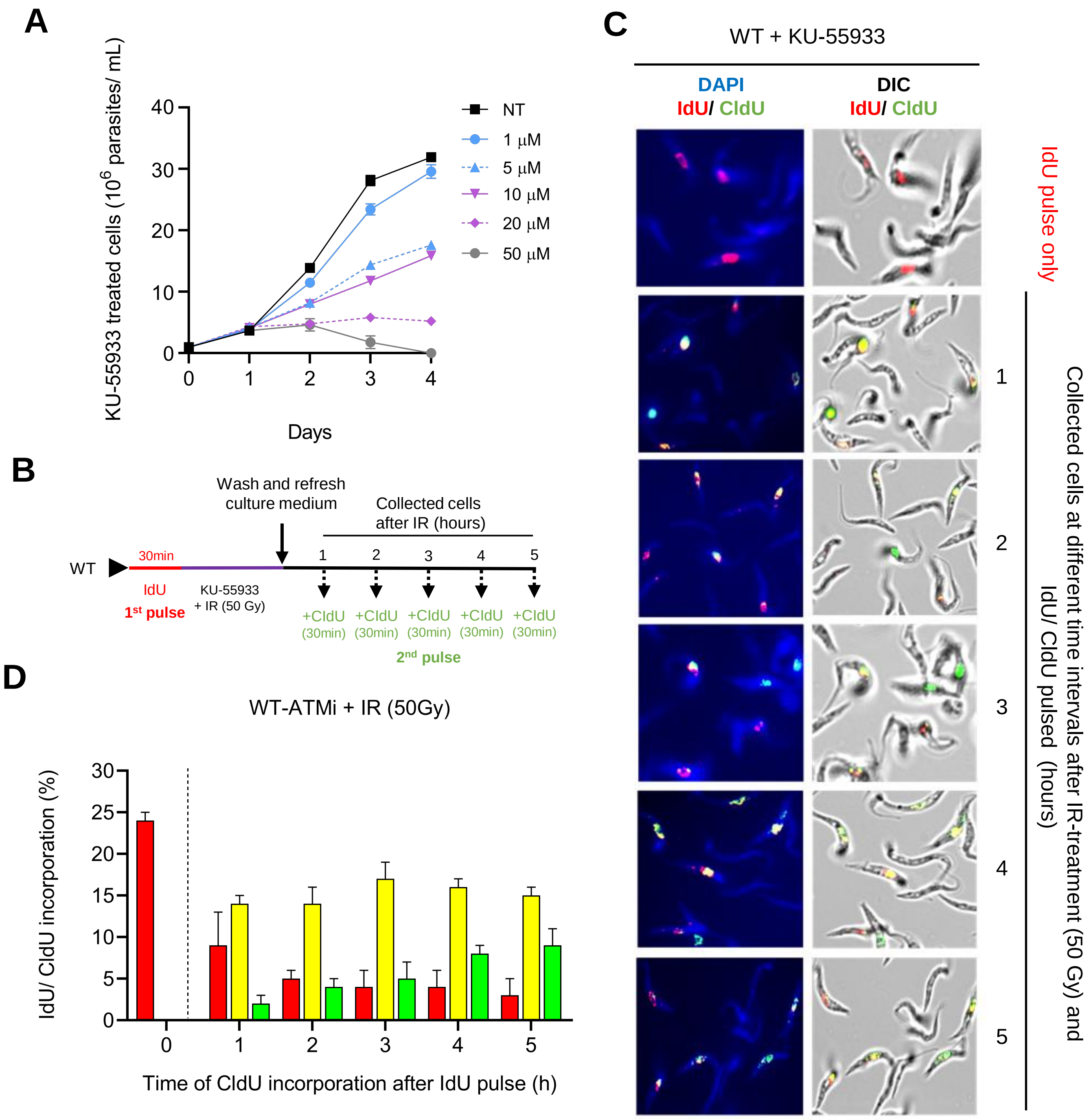

Supplementary Figure S4. Functions of ATM kinase in cell progression through the S phase after IR-irradiation. (A). Graph shows cell growth of the procyclic forms of *T. brucei* after exposure to different concentrations of the ATM inhibitor KU-55933 (1 - 50 μM) over time. The data represent the average of three independent experiments, and the bars represent the standard deviations. NT, not treated. **(B)** Experimental strategy for detection of cells that transit through the S phase in conditions of ATM inhibition and IR-irradiation. WT population was exposed to IdU for 30 min. Then, the cells were irradiated and cultured in the presence of the ATM inhibitor KU-55933 (20 μM). After that, the cells were pulsed with CIdU for 30 min before each measurement time and collected at the indicated times. **(C)** Codetection of thymidine analogs IdU and CldU in treated cells with ATM inhibitor and IR-irradiation. **(D)** Quantification of the parasites that progress through the S phase in the ATM-inhibited population after IR irradiation. Bar plots show the percentage of parasites in each group (cells leaving, entering or in the intra-S phase) and kinetics of thymidine analogs incorporation over time after IR-irradiation. The data represent the average of three independent experiments, each consisting of n = 300, and the error bars represent the standard deviations.

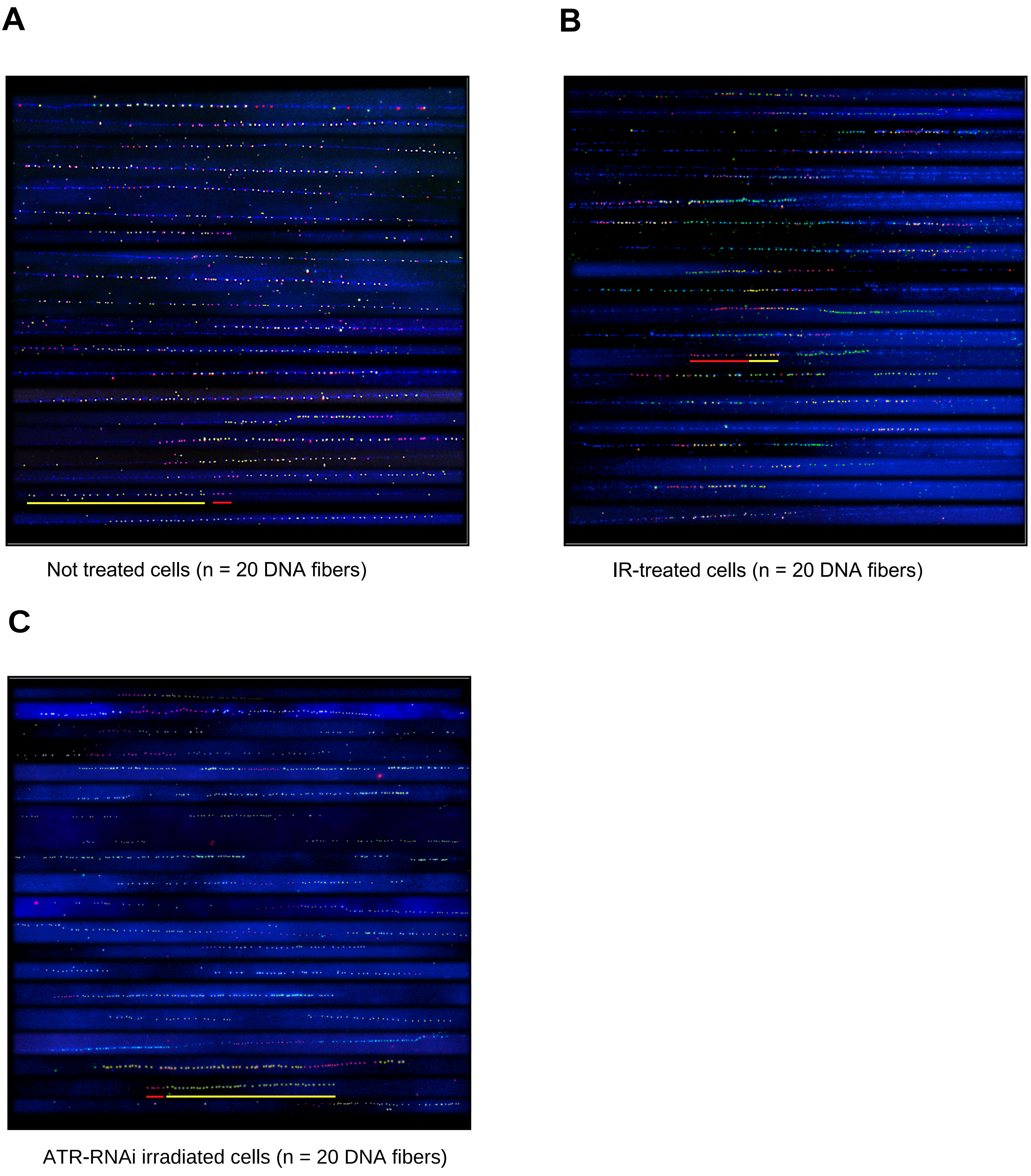

Supplementary Figure S5. DNA combing assay. (A-B) Panels of single-stranded DNA stretched from WT cells before and after IR irradiation. In **(B)**, the cells were irradiated after the first IdU pulse followed by a second CldU pulse. To assess the recovery of the replication fork, the cells were washed after the second pulse and pulsed again with CIdU 6 h after irradiation. **(C)** Panel of single-stranded DNA stretched from ATR-RNAi after IR irradiation. Each panel contains 20 DNA-stretched molecules used to estimate the length of the tracks of each of the analogs incorporated (IdU and CldU) and determine the DNA fork elongation factor (DFEF). The red and yellow bars represent the length of the one of tracks that was measured to calculatethe DFEF. The DFEF was determined as the ratio between the length of CldU incorporated after the 2nd pulse (yellow track) and the length of IdU incorporated after the 1st pulse (red track).

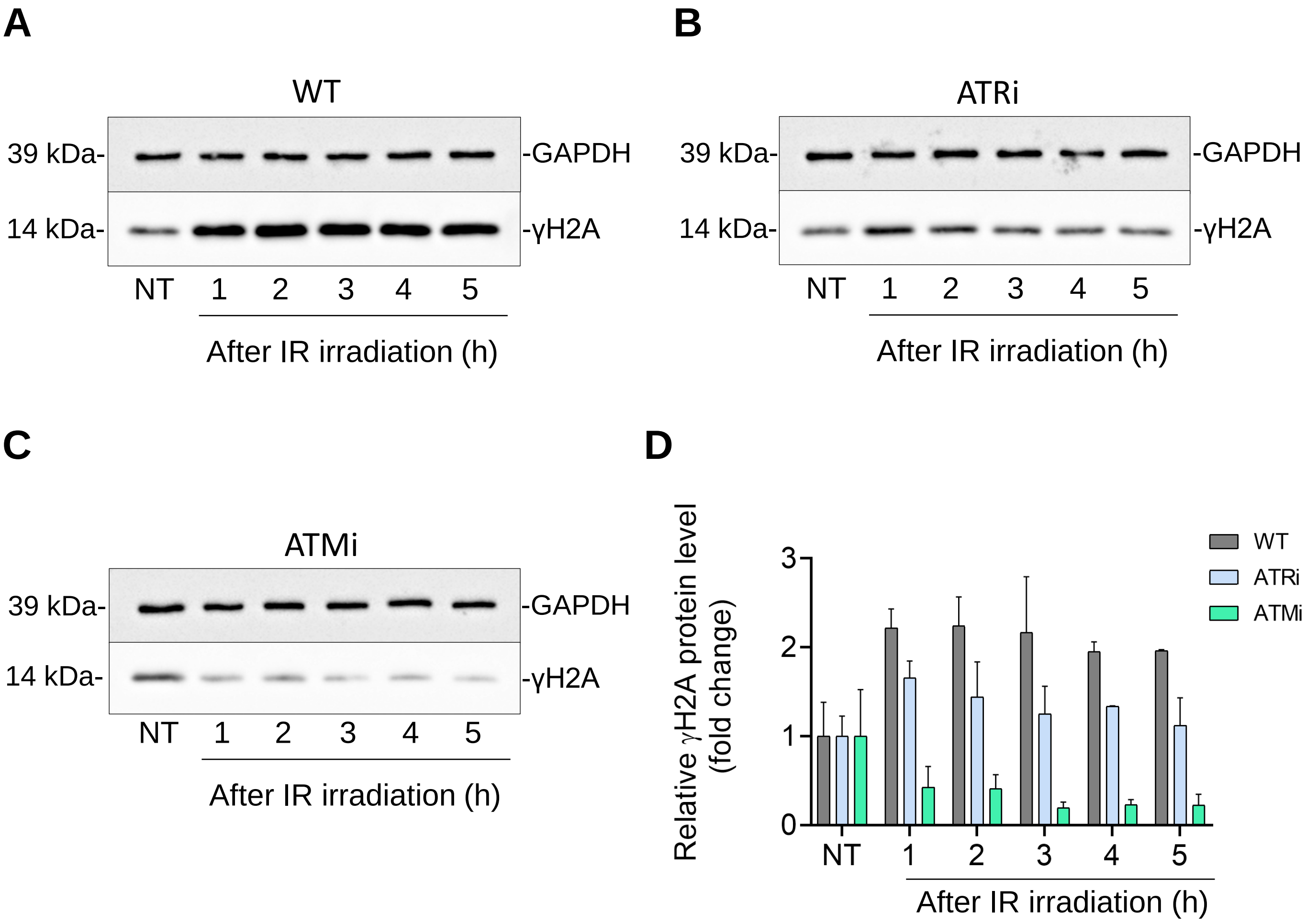

Supplementary Figure S6. Western blot analysis of γH2A protein level in WT, ATR or ATM inhibited cells after IR irradiation. The figure shows the γH2A protein level detected in *T. brucei* PCFs WT **(A)**, ATR inhibited cells **(B)** and ATM inhibited cells **(C)** after 50 Gy of IR irradiation. The *T. brucei* PCF were cultured in absence (WT) or in presence of 5 μM of ATR inhibitor VE-821 (ATRi) or 20 μM of ATM inhibitor KU-55933 (ATMi) and immediately treated with 50 Gy IR irradiation. The γH2A protein level was detected hourly up to 5 h after IR irradiation. **(D)** Quantification of the relative intensity of γH2A protein level in WT **(A)**, ATR inhibited cells **(B)** and ATM inhibited cells **(C)** after IR irradiation. The data represents the average of two independent experiments, and error bars represent the standard deviation. The Anti-GAPDH antibody was used as loading control. NT, not treated.
